# Polyphosphate: A Multifunctional Metabolite in Cyanobacteria and Algae

**DOI:** 10.3389/fpls.2020.00938

**Published:** 2020-06-26

**Authors:** Emanuel Sanz-Luque, Devaki Bhaya, Arthur R. Grossman

**Affiliations:** ^1^ Department of Plant Biology, The Carnegie Institution for Science, Stanford, CA, United States; ^2^ Department of Biochemistry and Molecular Biology, University of Cordoba, Cordoba, Spain

**Keywords:** polyphosphate, microalgae, cyanobacteria, acidocalcisome, stress responses, phosphate metabolism, metal toxicity, bioremediation

## Abstract

Polyphosphate (polyP), a polymer of orthophosphate (PO_4_^3-^) of varying lengths, has been identified in all kingdoms of life. It can serve as a source of chemical bond energy (phosphoanhydride bond) that may have been used by biological systems prior to the evolution of ATP. Intracellular polyP is mainly stored as granules in specific vacuoles called acidocalcisomes, and its synthesis and accumulation appear to impact a myriad of cellular functions. It serves as a reservoir for inorganic PO_4_^3-^ and an energy source for fueling cellular metabolism, participates in maintaining adenylate and metal cation homeostasis, functions as a scaffold for sequestering cations, exhibits chaperone function, covalently binds to proteins to modify their activity, and enables normal acclimation of cells to stress conditions. PolyP also appears to have a role in symbiotic and parasitic associations, and in higher eukaryotes, low polyP levels seem to impact cancerous proliferation, apoptosis, procoagulant and proinflammatory responses and cause defects in TOR signaling. In this review, we discuss the metabolism, storage, and function of polyP in photosynthetic microbes, which mostly includes research on green algae and cyanobacteria. We focus on factors that impact polyP synthesis, specific enzymes required for its synthesis and degradation, sequestration of polyP in acidocalcisomes, its role in cellular energetics, acclimation processes, and metal homeostasis, and then transition to its potential applications for bioremediation and medical purposes.

## Introduction. A Brief Overview of Polyphosphate Biology

### Orthophosphates, the Building Blocks of Polyphosphate

Phosphorus (P), mostly in the form of inorganic or orthophosphate (PO_4_^3−^), is integral to metabolic processes as a functional component of many molecules in the cell; these molecules include nucleic acids, phospholipids, phosphoproteins, and metabolites in most catabolic and anabolic pathways and signaling molecules. PO_4_^3-^ is the dominant form of P in the Earth's crust with levels in the soils often between 0.5 and 1.5 mM, although much of it may be insoluble and limiting to the growth of organisms in both ecological and agricultural environments. Soil P is mostly derived from the weathering of PO_4_^3–^containing minerals, primarily apatite (Ca_5_(PO_4_)_3_OH). While often found associated with Ca^2+^ salts, PO_4_^3-^ is also occluded in insoluble Fe^2+/3+^ and Al^3+^ salts and adsorbs onto surfaces of soil particles or becomes esterified to organic molecules, many that cannot be directly assimilated by most organisms. Bioavailable PO_4_^3-^ can be rapidly taken up by microbes, algae, and plants, which in turn can be consumed by grazers. This P is often returned to the environment as organic phosphates upon excretion and as the organisms die and decay. PO_4_^3-^ can also form phosphonyl bonds (carbon-phosphorus) generating a group of compounds called phosphonates that are present in marine invertebrates and can be metabolized (and synthesized) by bacteria, both in the oceans and freshwater environments (Dyhrman et al., 2006; Adams et al., 2008; Ilikchyan et al., 2009; Villarreal-Chiu et al., 2012).

Many organisms can also polymerize PO_4_^3-^ into polyphosphate (polyP) chains, that are composed of three to hundreds of PO_4_^3-^ groups linked by the high-energy phosphoanhydride bonds, the same bond that allows ATP to assume the role of the energy currency in cells. These polymers are present in all kingdoms of life, from tiny prokaryotic organisms and archaebacteria to large mammals, (Kornberg et al., 1999; Rao et al., 2009), although plants do not appear to synthesize polyP (Zhu et al., 2020) and mostly store PO_4_^3-^ in phytate (inositol hexakisphosphate, InsP6) (Raboy, 2003; Kolozsvari et al., 2015; Lorenzo-Orts et al., 2020), a six-fold dihydrogenphosphate ester of inositol. PolyP was likely a significant component of the pre-biological Earth as it can be spontaneously formed as a consequence of volcanic and hydrothermal vent activities (Rao et al., 2009; Achbergerova and Nahalka, 2011). It can accumulate to very high intracellular concentrations in microbes, ranging from µM to mM, especially under stress conditions when PO_4_^3-^ is abundant.

### Detection and Quantification

There are several ways to monitor polyP: these include visualization by transmission electron microscopy (Jensen, 1968) that can be coupled with spatially resolved elemental analysis (Eixler et al., 2005; Shebanova et al., 2017); phase contrast or bright-field microscopy after staining the cells with basic dyes including toluidine blue, methylene blue, and neutral red (Kulaev et al., 2004); binding polyP to the fluorochrome 4′,6-diamidino-2- phenylindole (DAPI); ^31^P nuclear magnetic resonance spectroscopy and determination of released PO_4_^3^ from polyP by the malachite green assay, which involve extraction and hydrolysis of the polyP (Beauvoit et al., 1989; Castro et al., 1995; Chen, 1999; Diaz et al., 2008; Khoshmanesh et al., 2012); and 2-dimensional Raman microscopy (Moudrikova et al., 2017). In photosynthetic organisms, some of these techniques can be difficult to optimize. For example, visualization of polyP can be obscured by pigmentation in photosynthetic cells; basic dyes can also bind nucleic acids and polyhydroxybutyrate (common components in cyanobacterial and algal cells) (Martinez, 1963; Kulaev et al., 2004), and ^31^P NMR (Hupfer et al., 2004; Hupfer et al., 2008; Kizewski et al., 2011) only detects P-containing molecules on the basis of bond class, the presence of other molecules with phosphoanhydride bonds (*e.g.* nucleotides) may cause inaccuracies in measurements, especially if these molecules are abundant. DAPI is one of the most commonly used reagents to identify polyP (polyP binding to the fluorophore alters its peak of fluorescence) (Tijssen et al., 1982; Aschar-Sobbi et al., 2008). It is a simple, inexpensive molecule that allows visualization and quantification of polyP in cells (Gomes et al., 2013; Martin and Van Mooy, 2013), although DAPI can also bind nucleic acids and inositol polyphosphate (Kolozsvari et al., 2014), making treatment with RNase and DNase (Martin and Van Mooy, 2013; Martin and Van Mooy, 2015) and optimization of protocols necessary to increase the quantification accuracy (Bru et al., 2016).

### Metabolism and Storage

In prokaryotes and some eukaryotes, including *Dyctiostelium discoideum*, the synthesis of polyP is mediated by PolyP Kinase (PPK) (Brown and Kornberg, 2004; Brown and Kornberg, 2008; Hooley et al., 2008; Livermore et al., 2016a; Weerasekara et al., 2016; Blaby-Haas and Merchant, 2017), while in most eukaryotes (fungi, protists, and algae) its synthesis requires polyP polymerase activity of VTC4. This enzyme, which is part of the Vacuolar Transporter Chaperone (VTC) complex (Hothorn et al., 2009; Aksoy et al., 2014; Ulrich et al., 2014; Desfougeres et al., 2016; Gerasimaite and Mayer, 2016; Blaby-Haas and Merchant, 2017; Gomes-Vieira et al., 2018), has no evolutionary relationship to PPK. Protein(s) responsible for the synthesis of polyP in animals has not, at this point, been identified. The catalytic reaction for both the prokaryotic and eukaryotic polyP synthesizing enzymes involves the transfer of the terminal PO_4_^3-^ of ATP to the growing polyP chain (although PPK can also use 1,3-diphosphoglycerate). The synthesis of polyP in prokaryotes is under *pho* regulatory control (Santos-Beneit, 2015), while, in eukaryotic organisms, polyP synthesis is linked to inositol phosphate (InsP) metabolism (Auesukaree et al., 2005; Lonetti et al., 2011; Ghosh et al., 2013; Wild et al., 2016; Cordeiro et al., 2017; Gerasimaite et al., 2017). Inositol phosphates are signaling molecules synthesized from glucose through a pathway that is conserved from Archaea to humans. These molecules perform a wide variety of functions and are linked to P and ATP cellular homeostasis (Saiardi, 2012; Azevedo and Saiardi, 2017).

PO_4_^3-^ can be mobilized from polyP through the catalytic activity of enzymes that degrade the polymer, including both endo- and exo-polyphosphatases (Akiyama et al., 1993; Kornberg et al., 1999; Rodrigues et al., 2002; Fang et al., 2007b; Lichko et al., 2010). Pyrophosphate generated during polyP degradation can be used as a source of energy (like polyP and ATP) by various organisms (Lahti, 1983), but can also be further hydrolyzed by a vacuolar/acidocalcisome soluble pyrophosphatase without conserving the phosphoanhydride bond energy (Lemercier et al., 2004; Huang et al., 2018).

The main site of synthesis and storage of polyP is the acidic vacuole designated the acidocalcisome, discovered more than 100 years ago (Meyer, 1904; Vercesi et al., 1994) based on their visual prominence because they house densely stained polyP granules (Docampo et al., 2005; Miranda et al., 2008; Docampo, 2016; Docampo and Huang, 2016). These vacuoles have been characterized in disease causing trypanosomatids and apicomplexan parasites (Luo et al., 2004; Ruiz et al., 2004b; Fang et al., 2007a; Moreno and Docampo, 2009; Madeira da Silva and Beverley, 2010; Li and He, 2014; Kohl et al., 2018) and algae (Aksoy et al., 2014; Goodenough et al., 2019), and have similar characteristics to vacuoles in fungi (Gerasimaite et al., 2017) and animal cells (Ruiz et al., 2004a; Huizing et al., 2008; Muller et al., 2009; Moreno-Sanchez et al., 2012; Morrissey, 2012). However, there may also be acid-soluble polyP pools in other cellular locations (*e.g*. bacterial cell walls). Acidocalcisomes can attain polyP levels of 3–8 M (Moreno et al., 2002), accumulate Ca^2+^ and other divalent cations, as well as some organic molecules (Kaska et al., 1985; Vercesi et al., 1994; Siderius et al., 1996; Komine et al., 2000; Ruiz et al., 2001; Docampo and Moreno, 2011; Hong-Hermesdorf et al., 2014; Penen et al., 2016; Klompmaker et al., 2017; Penen et al., 2017; Steinmann et al., 2017; Tsednee et al., 2019). In trypanosomes, these vacuoles acidify the lumen down to pH ~5.0 using both the ubiquitous V-type ATPase and the H^+^-PPase proton pump (Scott and Docampo, 2000), although the latter may not be present in animal and yeast “acidocalcisomes.” In lower eukaryotes, the VTC complex (Cohen et al., 1999; Hothorn et al., 2009; Gerasimaite et al., 2014) is located on the acidocalcisome membrane, anchored by a region of the VTC subunits containing three transmembrane domains (designated VTC domain).

### Overview of Functions

A lack of appreciation of the importance of polyP metabolism over the last several decades has caused some researchers to refer to this polymer as a “molecular fossil” (Kornberg and Fraley, 2000; Manganelli, 2007), although, an increasing number of studies implicate polyP in a variety of processes. It can either directly or indirectly buffer changes in cellular PO_4_^3-^ and adenylate levels, which are critical since elevated intracellular PO_4_^3-^ and ATP levels can inhibit many cellular reactions (*e.g.* reversible reactions in which PO_4_^3-^ is an end-product). PolyP also provides a reservoir of chemical bond energy for driving biological processes, although it may not be the most efficient source of energy because of its slow metabolic turnover rate relative to ATP (Van Mooy et al., 2009). The anionic nature of polyP enables it to bind and sequester cations, which can contribute to the tolerance of cells to heavy metals, preventing metabolic aberrations resulting from elevated intracellular cation concentrations (van Groenestijn et al., 1988; Rao and Kornberg, 1999; Andreeva et al., 2014), serve as a chaperone (Gray et al., 2014; Xie and Jakob, 2018) and a structural/functional component in membrane ion channels (Reusch, 1999; Reusch, 2000). In some cases, polyP can also modify protein function through post-translational attachment to lysine residues in a process known as polyphosphorylation (Azevedo et al., 2015). Organisms/cells with defects in the synthesis of polyP exhibit a range of abnormalities including cancerous proliferations, defects in cellular signaling (including TOR signaling), an inability to normally perform certain cellular processes such as autophagy and apoptosis, biofilm formation, sporulation, quorum sensing, pathogen virulence, and acclimation to both biotic and abiotic stresses, including stationary phase survival and nutrient deprivation (Kornberg et al., 1999; Rao and Kornberg, 1999; Rashid et al., 2000; Shi et al., 2004; Fraley et al., 2007; Diaz-Troya et al., 2008; Rao et al., 2009; Aksoy et al., 2014; Li and He, 2014). PolyP can also be released from acidocalcisomes of human platelets to modulate clotting and fibrinolysis (Ruiz et al., 2004a; Smith et al., 2006). Moreover, there is a growing realization of the importance of polyP with respect to cell physiology and biogeochemical cycling in marine ecosystems, as indicated by both large scale environmental analyses (Diaz et al., 2008; Orchard et al., 2010; Martin et al., 2014; Diaz et al., 2016; Dijkstra et al., 2018; Martin et al., 2018) and laboratory studies (Rhee, 1973; Jensen and Sicko-Goad, 1976; Jacobson et al., 1982; Orchard et al., 2010; Martin et al., 2014; Diaz et al., 2016). Even though the precise mechanisms by which polyP synthesis and accumulation impact cellular processes may sometimes be uncertain, it is clearly a functional giant with an impressive resume!

In this review, we highlight major aspects of polyP metabolism, storage, and function in photosynthetic organisms. However, throughout the text we refer the reader to several other recent reviews that cover the biology of polyP (Kornberg, 1995; Kornberg et al., 1999; Kulaev et al., 2004; Docampo et al., 2005; Rao et al., 2009; Docampo and Moreno, 2011; Kulakovskaya et al., 2012; Docampo, 2016; Livermore et al., 2016b; Jimenez et al., 2017; Xie and Jakob, 2018).

## PolyP Synthesis, Localization, and Storage in Photosynthetic Microbes

Cyanobacteria and algae are photosynthetic organisms that serve as primary producers in terrestrial, marine, and freshwater habitats (Waterbury et al., 1979; Weisse, 1993; Sliwinska-Wilczewska et al., 2018). These organisms can survive very harsh environmental conditions, including those of the hot spring microbial mats (Ward et al., 1998) and the biofilms that form the sand crusts of deserts (Treves et al., 2013; Treves et al., 2016; Oren et al., 2019). Cyanobacteria and algae are also prominent components in dense bacterial blooms that can cause eutrophication of water bodies and release toxins into the environment (Wang, 2008; Anderson et al., 2012; Jasser and Callieri, 2017; Sliwinska-Wilczewska et al., 2018; Syed Hasnain et al., 2019), potentially compromising potable water resources. The productivity of photosynthetic microbes is often constrained by the availability of nutrients, including PO_4_^3-^, and like other unicellular organisms they can store large amount of polyP in granules called polyP bodies (PPBs) which are often found in acidocalcisomes (Jensen and Sicko-Goad, 1976; Grillo and Gibson, 1979; Gomez-Garcia et al., 2003; Gomez-Garcia et al., 2013; Aksoy et al., 2014; Goodenough et al., 2019).

### Regulation of PolyP Metabolism

Cyanobacterial genes encoding enzymes involved in the synthesis (polyphosphate kinase, *ppk*) and degradation (exo-polyphosphatase, *ppx*) of polyP have been identified through the generation of mutants (Gomez-Garcia et al., 2003; Gomez-Garcia et al., 2013). However, there is still limited knowledge concerning the mechanisms involved in regulating polyP metabolism in cyanobacteria (Jensen and Sicko-Goad, 1976; Grillo and Gibson, 1979). Levels of *ppk* and *ppx* transcripts have been measured for a strain of *Synechococcus* present in hot spring microbial mats (growing at 50–60°C) of Yellowstone National Park. *In situ* measurements demonstrated that the *ppk* and *ppx* transcript and polypeptide levels varied over the diel cycle (Gomez-Garcia et al., 2003), as was also noted for components of other pathways including those associated with photosynthesis, respiration, nitrogen fixation, fermentation, and oxidative stress (Steunou et al., 2006; Steunou et al., 2008). While the level of the *ppk* mRNA peaked at night, *ppx* mRNA accumulation was highest during the early morning. It was hypothesized that the pattern of diel accumulation observed for these transcripts/enzymes could account for polyP storage and accumulation in cyanobacteria of the hot springs and participate in coordinating daily growth and energy demands during the light period with PO_4_^3-^ utilization. While we still have little detailed knowledge of regulatory factors impacting *ppk* transcription and/or PPK activity, mutants of *Anacystis nidulans* (now *Synechococcus*) with decreased PO_4_^3-^ assimilation and polyP accumulation were generated by treating cells with ethyl methanesulfonate (EMS) or *N*-methyl nitrosoguanidine (NTG); based on TEM, these mutants often exhibited a loss of polyP granules and were impacted in their level of PPK activity, either because of a change in the activity of the enzyme or because of altered *ppk* expression (Vaillancourt et al., 1978). On the other hand, transcripts encoding Ppx and Ppa (inorganic pyrophosphatase) proteins, involved in polyP and pyrophosphate degradation, respectively, increased upon long term PO_4_^3-^ deprivation of the cyanobacterium *Synechocystis* PCC6803 (Gomez-Garcia et al., 2003).

The Pho regulon controls most genes associated with the acclimation to PO_4_^3-^ deprivation in bacteria. In *Synechocystis* PCC6803, the major controlling elements of this regulon are SphS (sll0337; histidine kinase; analogous to *E. coli* PhoR) and SphR (slr0081; response regulator; analogous to *E. coli* PhoB) (Hirani et al., 2001; Suzuki et al., 2004; Juntarajumnong et al., 2007). The regulation of genes encoding the enzymes that degrade polyP appear to be under the control of PhoU, a negative regulator of the Pho regulon (causes suppression of the Pho regulon activity under P-replete conditions) (Wanner, 1993; Morohoshi et al., 2002). There are also some works suggesting that the concentrations of (p)ppGpp, a stringent response second messenger, can stimulate polyP accumulation in *E. coli* (Kuroda et al., 1997) and is potentially also involved in polyP accumulation in *Synechococcus* in the dark (Seki et al., 2014; Hood et al., 2016). Hence, while some specific elements have been shown to contribute to the control of the synthesis and degradation of polyP in cyanobacteria, the details of this control have not been examined.

**Figure 1:**
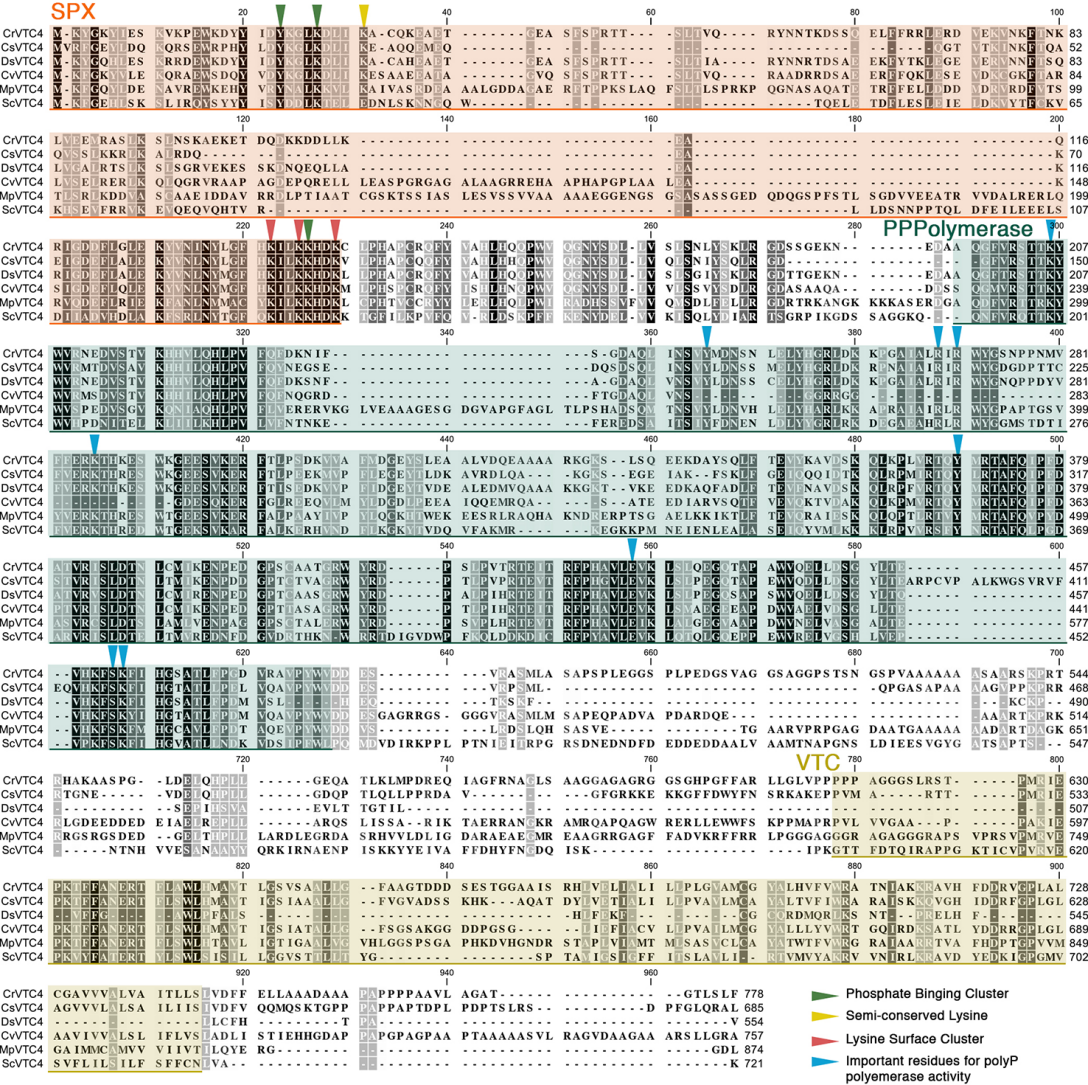
Alignment of microalgal VTC4 proteins. Protein sequences of *Chlamydomonas reinhardtii* (CrVTC4, PNW79162.1), *Coccomyxa subellipsoidea* (CsVTC4, XP_005646401.1)*, Dunaliella salina* (DsVTC4, Dusal.0567s00005.1)*, Chlorella variabilis* (CvVTC4, XP_005844713.1)*, Micromonas pusilla* (MpVTC4, XP_003055174.1), and *Saccharomyces cerevisiae* (ScVTC4, NP_012522.2) were aligned using the CLC Sequence Viewer software. SPX, polyP polymerase, and VTC domains are highlighted in orange, green, and yellow shaded boxes, respectively. Inverted triangles indicate functional residues previously described in yeast.

In unicellular algae, the synthesis of polyP has not been characterized in detail, although VTC proteins are conserved in a variety of algal species. In the green, unicellular alga *Chlamydomonas reinhardtii* (*C. reinhardtii* throughout), a mutant lacking VTC1 is unable to accumulate polyP (Aksoy et al., 2014), which indicates that the VTC complex is likely required for the synthesis of polyP in this alga. The VTC4 protein, the subunit that catalyzes polyP synthesis and its translocation into the acidocalcisomes in yeast, has not yet been studied in photosynthetic organisms, but the gene encoding this protein is present in the genome of many algal species. As in budding yeast and *Trypanosoma*, the algal VTC4 proteins have the SPX, PolyP Polymerase, and VTC domains, and all of the key residues related to VTC4 functionality are conserved (**Figure 1**). Therefore, it seems reasonable to hypothesize that these VTC4 proteins catalyze polyP synthesis and are activated by the binding of inositol pyrophosphates (most phosphorylated forms of inositol) to their SPX domains, as in other eukaryotes (Wild et al., 2016; Gerasimaite et al., 2017).

### Acidocalcisomes and the Synthesis and Storage of Polyphosphate

Generally, polyP accumulates in acidocalcisomes in unicellular eukaryotes, although it can be present in other cellular compartments including the nucleus, mitochondria, cytoplasm, cell wall, and endoplasmic reticulum. In cyanobacteria, polyP granules have been observed in the center of the cell and in close proximity to carboxysomes (Nierzwicki-Bauer et al., 1983; Liberton et al., 2011), the cellular organelle that houses ribulose-1,5-bisphosphate carboxylase which is critical for the fixation of inorganic carbon. Cyanobacteria have also been observed to have polyP in regions of the cell containing ribosomes, in close association with DNA, and in the intrathylakoid space (Jensen and Sicko, 1974). In algae, several studies report polyP accumulation in acidocalcisomes (Komine et al., 2000; Ruiz et al., 2001; Aksoy et al., 2014; Goodenough et al., 2019). *C. reinhardtii* acidocalcisomes have been examined by TEM (Heuser, 2011) and in detail after freeze-fracture, deep-etching, and platinum rotary-replication (Goodenough et al., 2019). Algal acidocalcisomes have been described as *de novo* assembled vacuoles in the trans-Golgi that show diverse variants according to their disposition, composition, and consistency (Goodenough et al., 2019). However, very little is known about the physiological relevance of these observed variations. The number and size of polyP bodies in cyanobacterial and algal cells can vary greatly, but they generally increase under stress conditions (Stevens et al., 1985; Jensen, 1993; Docampo et al., 2010; Aksoy et al., 2014; Tsednee et al., 2019). As in other single-cell eukaryotes, the acidocalcisome membranes in *C. reinhardtii* harbor the VTC complex, which is responsible for the polyP polymerase and H^+^-PPase activities (acidocalcisome membranes also harbor a V-type ATPase driven proton pump); the H^+^-PPases, with homologues in three eukaryotic clades (not present in opisthokonts and Amoebozoa), are activated by pyrophosphate (Rea et al., 1992; Kim et al., 1994; Rodrigues et al., 1999; McIntosh and Vaidya, 2002; Drozdowicz et al., 2003; Au et al., 2006; Serrano et al., 2007; Hsu et al., 2009; Tsai et al., 2014; Schilling et al., 2017; Shah et al., 2017; Segami et al., 2018a; Segami et al., 2018b) and also function in acidification of the lumen of vacuoles in land plants (Venter et al., 2006; Kajander et al., 2013; Asaoka et al., 2014).

Acidocalcisomes have been isolated from the red alga *Cyanidioschyzon merolae* and kinetoplastids and their proteome has been characterized (Yagisawa et al., 2009; Huang et al., 2014). They harbor hydrolytic enzymes, components of vesicular trafficking pathways (Rabs, SNAREs) that are associated with their biogenesis (Besteiro et al., 2008; Huang et al., 2011; Li and He, 2014; Niyogi et al., 2015) and aquaporins (Montalvetti et al., 2004). Additionally, it was recently shown that inositol pyrophosphates regulation (Cordeiro et al., 2017) plays an important role in acidocalcisome function (Huang et al., 2013; Lander et al., 2013), participating in Ca^2+^ homeostasis through activation of Ca^2+^ channels based on work with trypanosomatids [reviewed in (Ramakrishnan and Docampo, 2018)].

PolyP has also been localized in the cell wall of different algal species (*C. reinhardtii*, *Volvox aureus*, and *Coleochaete scutata*) (Werner et al., 2007). This was determined based on binding to a polyP binding protein (*E. coli* exopolyphosphatase, EcPPX) coupled with detection by immunofluorescence using antibodies against a maltose-binding protein fused to the EcPPX. PolyP accumulation was observed during mitosis, with a strong signal at the end of cytokinesis, and in *C. reinhardtii*, accumulation appeared highest in the mother cell envelope following mitosis and just before the release of the daughter cells. These daughter cells maintained the polyP in their cell wall for a short time after being released. The role of cell wall polyP is not clear, but it may have a protective function during cytokinesis when the daughter cell walls are not fully formed and serve as a barrier to toxic agents (*e.g.* pathogens or heavy metals; see section 3.4) during this ‘vulnerable' period (Werner et al., 2007; Penen et al., 2017).

## PolyP Functions and Regulation in Photosynthetic Microbes

### PolyP as PO_4_^3-^ Reservoir During P Deprivation

**Figure 2:**
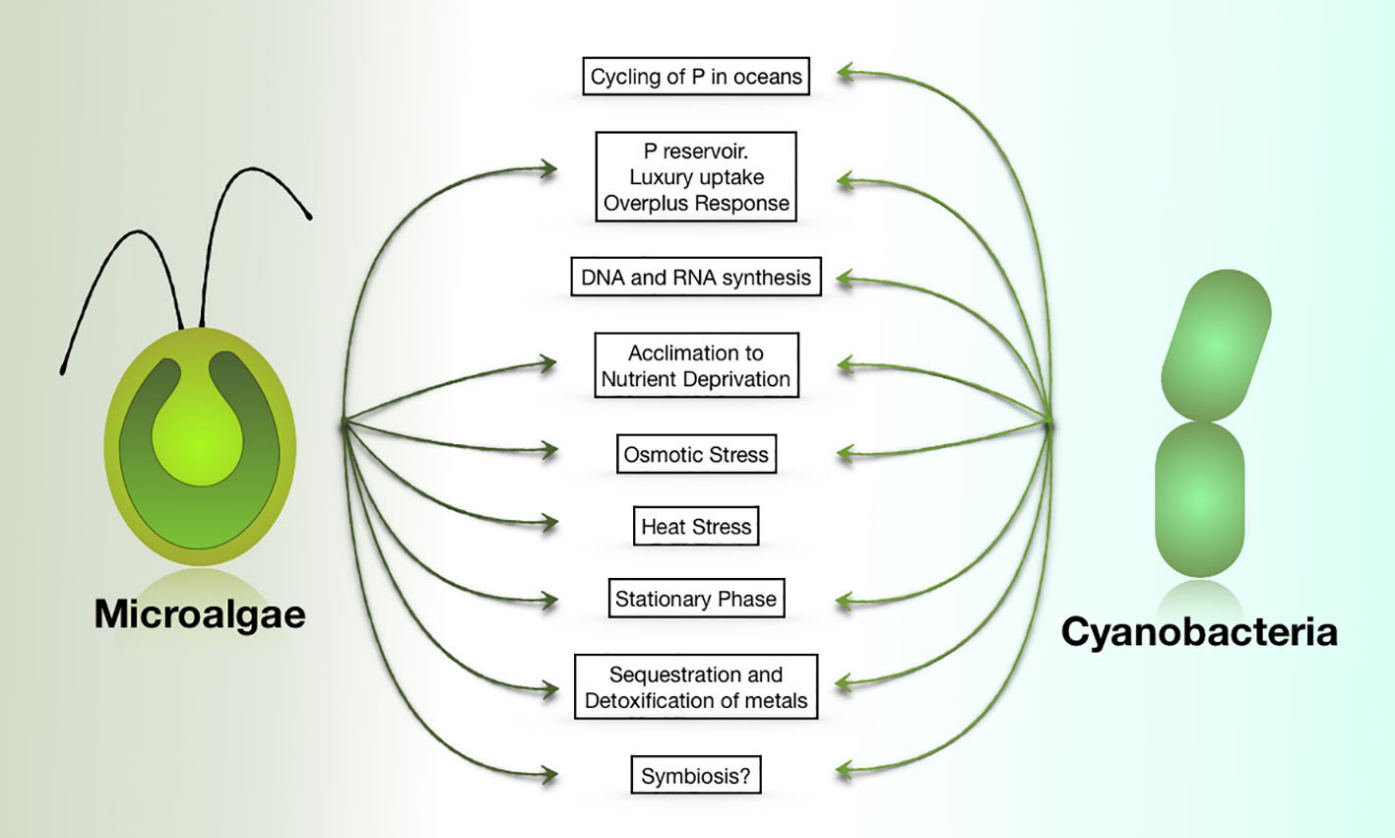
PolyP functions in cyanobacteria and eukaryotic microalgae. Most of these functions have also been ascribed to polyP in non-photosynthetic organisms.

Various functions and cellular responses are impacted by the presence/synthesis of polyP (**Figure 2**). Several studies have suggested that when the supply of PO_4_^3-^ is limiting in the environment, including in the sparse nutrient environments of the subtropical gyres (Perry, 1976), polyP functions as a dynamic PO_4_^3-^ reservoir (Kornberg et al., 1956; Harold, 1966)). “Sparing” (limiting the use of a molecule in cellular processes when it is not readily available) and internal scavenging of PO_4_^3-^ from polyP, nucleic acids, and phospholipids allow PO_4_^3-^ redistribution to accommodate processes that may extend survival during P deprivation. The presence/absence of polyP granules are indicators of the P status of organisms, but other indicators such as high affinity PO_4_^3-^ uptake, extracellular alkaline phosphatase activity, and the replacement of phospholipids with sulfolipids in cyanobacteria and eukaryotic algae have been reported (Dyhrman and Palenik, 1999; Riegman et al., 2000; Karl and Bjorkman, 2002; Hoppe, 2003; Dyhrman and Ruttenberg, 2006; Moseley and Grossman, 2009; Van Mooy et al., 2009; Bar-Yosef et al., 2010; Harke et al., 2012; Reistetter et al., 2013; Munoz-Martin et al., 2014; Wan et al., 2019).

In both prokaryotic and eukaryotic microbes, the role of polyP as a PO_4_^3-^ reservoir depends on the organism's capacity to take up PO_4_^3-^, synthesize and store polyP, especially when PO_4_^3-^ is more abundant than is required for growth, and degrade it when the level of available PO_4_^3-^ in the environment becomes limiting (Orchard et al., 2010). This accumulation dramatically increases when organisms are preconditioned in medium deficient for P. Exposure to excess PO_4_^3-^ after this preconditioning leads to the “luxury uptake” of PO_4_^3-^, enhanced polyP synthesis and accumulation in acidocalcisomes, which can serve as P and energy reservoirs. This phenomenon has been designated the “overplus” or over-compensation response and has been extensively studied in cyanobacteria (phytoplankton) and algae in ocean, lake, and river habitats (Liss and Langen, 1962; Harold, 1963; Harold, 1964; Voelz et al., 1966; Aitchison and Butt, 1973; Sicko-Goad and Jensen, 1976; Grillo and Gibson, 1979; Tyrell, 1999; Karl and Bjorkman, 2002; Hoppe, 2003; Kulaev et al., 2004; Hupfer et al., 2007; Falkner and Falkner, 2011). The accumulation of polyP during luxury uptake is considered important for protection of aquatic organisms against future exposure to P limitation (Hupfer et al., 2004; McMahon and Read, 2013). For *Synechocystis* PCC6803, PO_4_^3-^ uptake and polyP accumulation by cells exposed to overplus conditions resulted in the initial detection of polyP granules in most cells within 3 min of replenishing the medium with PO_4_^3-^, with the number of cells containing these granules sustained for about 1 h (Voronkov and Sinetova, 2019) followed by degradation and redistributed of the polyP within the cell over a period of a few to several days. Additionally, polyP accumulation in *Synechocystis* occurred to a lesser extent in the dark than in the light and did not appear to be very sensitive to temperature changes. The overplus response and accumulation of polyP may be an adaptation to fluctuations of PO_4_^3-^ in the natural environment, where the levels of polyP can vary dramatically over short time periods; such a response would help sustain growth and viability even under periods of low PO_4_^3-^ availability (Droop, 1973; Falkner and Falkner, 2003; Hupfer et al., 2004; Falkner and Falkner, 2011; McMahon and Read, 2013). However, there is variability in the capabilities of cyanobacteria to accumulate polyP and reach a PO_4_^3-^ threshold at which polyP reserves are preferentially degraded, which may reflect the environment in which they evolved. In the filamentous bloom forming cyanobacterium *Nodularia spumigena*, vegetative cells exhibited different polyP levels than heterocysts (Braun et al., 2018). When PO_4_^3-^ is added back to P-depleted cultures, the PO_4_^3-^ is taken up from the medium and polyP accumulates preferentially in the vegetative cells. However, intracellular P in heterocysts remained low, highlighting functional differences between heterocysts and vegetative cells, in addition to their ability to fix molecular nitrogen.

Algae also exhibit the over-plus phenomenon. Two species of *Chlamydomonas* (*C. acidophila* KT-1 and *C. reinhardtii* C-9) exhibited the same pattern of polyP degradation when deprived of P, and the over-plus response was observed when the medium of the P-deprived cells was supplemented with an abundance of PO_4_^3-^. However, the levels of total polyP that accumulated varied between these species (Nishikawa et al., 2006). The overplus effect in the green alga *Chlorella vulgaris* depended on the duration of the starvation period and the PO_4_^3-^ concentration used during re-supply of the nutrient, with maximum accumulation after more than 8 h of deprivation and with PO_4_^3-^ resupply concentrations above 0.3 mM (Aitchison and Butt, 1973). So far, the specific factors that control the “overplus” response are not well understood. However, in eukaryotic algae, where the VTC complex is responsible for the synthesis of polyP, the binding of inositol pyrophosphates to the SPX domain of VTC4 must be considered since it is required to induce polyP polymerase activity in yeast (Wild et al., 2016; Gerasimaite et al., 2017). Following PO_4_^3-^ replenishment, accumulation of inositol pyrophosphates may be the first strategy for storing PO_4_^3-^ and polyP would be synthesized only when the cellular P or ATP concentrations are elevated to a level that triggers increased inositol pyrophosphate synthesis and binding to the SPX domains of the VTC polypeptides. A better understanding of this process, which has the potential to impact biotechnological strategies being developed for improved recovery of PO_4_^3-^ from wastewater, is needed (see *PolyP and Biotechnological Applications*).

The over-compensation phenomenon has also been studied in the red macroalga *Chondrus crispus.* After starving this alga for 2 weeks, it was resupplied with PO_4_^3-^ at a concentration that was greater than twice the concentration required to saturate the alga's P requirement (15 µM) (Chopin et al., 1997), however polyP accumulation was not observed. This finding suggests that over-compensation works differently or occurs under more extreme PO_4_^3-^conditions (*e.g*. more extended starvation, higher PO_4_^3-^concentration during resupply) or that different acclimation processes in response to changes in environmental PO_4_^3-^concenrations occur in multicellular photosynthetic organisms.

The presence of stored polyP in cells usually indicates a high PO_4_^3-^ concentration in the environment and the occurrence of luxury uptake, or a spike in the level of intracellular nutrients resulting from over-plus uptake (Karl, 2002; Karl and Bjorkman, 2002; Karl, 2014). However, the use of more sensitive methods for detection indicated that polyP was common in some marine environments, even when extracellular levels of P were low (Diaz et al., 2008; Diaz and Ingall, 2010; Orchard et al., 2010; Martin and Van Mooy, 2013). In phytoplankton populations extending from the western North Atlantic, the Gulf Stream, and into the severely P limited Sargasso Sea, it was demonstrated that P pools declined and two bio-indicators of low P conditions, alkaline phosphatase activity and the sulfolipid:phospholipid ratio, were both elevated in the biota. The lowest P concentration was measured in surface waters where the absorption of sunlight stimulated phytoplankton productivity. Surprisingly, as the level of inorganic P declined, the proportion of polyP with respect to total particulate P increased, indicating that a greater proportion of the P pool in the cell was maintained as polyP under conditions in which the level of inorganic P was at its lowest. This result was also noted specifically for cultures of marine *Synechococcus*, an abundant taxon in the oligotrophic oceans (Bjorkman, 2014; Martin et al., 2014). Overall, studies of Sargasso Sea phytoplankton conflict with the notion that polyP only serves as a luxury P reservoir that is rapidly mobilized when inorganic P levels in the environment decline. Furthermore, in the Sargasso Sea polyP was released from cells preferentially over bulk P, helping to sustain P levels in shallow waters, the zone of greatest metabolic activity. This suggests that polyP cycling in the environment may form a feedback loop that attenuates P export when environmental P becomes scarce and allows for the gradual contribution of bioavailable P to the ecosystem for sustaining primary production and supporting the use of exported carbon and nitrogen (Martin et al., 2014).

Studies with the diatom *Thallasiossira pseudonana* also demonstrated a preference for maintaining polyP even when the cells experience P deprivation. Gene expression and protein abundance patterns supported the observation that P deprivation of *T. pseudonana* resulted in an increased proportion of cellular P stored as polyP (Dyhrman et al., 2012). This observation is also in agreement with the general idea that not all polyP allocation is driven by luxury uptake and that controlled polyP cycling could be a key adaptation in low P ecosystems (Karl and Bjorkman, 2002). Similarly, the cyanobacterium *Microcystis aeruginosa,* is able to take up and store PO_4_^3-^ as polyP even when the concentration of external PO_4_^3-^ is low, which would enable it to compete with other phytoplankton when P becomes limiting (Wan et al., 2019). Taken together, these observations suggest that not all polyP allocation is driven by luxury uptake and that controlled polyP cycling could be a key component of the adaptation process in low P ecosystems (Karl and Bjorkman, 2002).

Although polyP is mainly stored in acidocalcisomes, as mentioned above, it can also be found in the cell wall, and associated with the cytoplasmic membrane and other macromolecules in the cell, which may reflect its varied functionalities. For example, it has been suggested that polyP can serve as a PO_4_^3-^ reservoir for the synthesis of DNA and RNA. *Synechococcus* sp. cells can contain multiple copies of genomic DNA (Binder and Chisholm, 1990; Binder and Chisholm, 1995; Mori et al., 1996). Some reports have noted an association of the fibrous structures in the nucleoid region of cyanobacterial cells with the PPBs (Voelz et al., 1966; Lawry and Jensen, 1979). There also appears to be a link between PPBs dynamics and chromosomal DNA behavior related to day-night cycling of cells. Cyanobacterial DNA synthesis occurs in the light and is dependent on photosynthetic electron transport (Binder and Chisholm, 1990; Watanabe et al., 2012; Ohbayashi et al., 2013; Seki et al., 2014), with cell elongation and division occurring for most cells toward the end of the light period (Seki et al., 2014). In cultures of the unicellular cyanobacterium *Synechococcus* that were synchronized to a light-dark cycle, DNA appeared diffuse during the dark period and exhibited transient compaction (based on fluorescence microscopy) at the end of the light period (Smith and Williams, 2006). This compacted DNA, observed by high-voltage cryo-electron tomography (Murata et al., 2016), forms structures visually similar to condensed eukaryotic chromosomes and appears to be associated with small, paired PPBs. Additionally, phase contrast transmission electron microscopy of *Synechococcus elongatus* PCC 7942 cells showed that newly synthesized BrdU (5-bromo-2'-deoxyuridine) labeled DNA was in close proximity to the PPBs (Nitta et al., 2009). The coordinated dynamics of PPBs/polyP levels, DNA morphology, and the observed interactions between these molecules over the period when DNA is being replicated, suggest that the PPBs supply PO_4_^3-^ for DNA replication in the light, and may participate in regulating DNA synthesis (Murata et al., 2016).

Another potential connection between polyP and DNA synthesis has been revealed by analyses of akinetes, spore-like cells that develop in some cyanobacteria that are specialized to survive adverse environmental conditions. The akinetes are generally larger than vegetative cells and differentiate from vegetative cells within the cyanobacterial filaments. They develop a thick cell wall as they mature and are dormant under harsh environmental conditions. When conditions improve, the akinetes divide and give rise to a new population of vegetative cells that can disperse in the environment (Hori et al., 2003; Karlsson-Elfgren and Brunberg, 2004; Karlsson-Elfgren et al., 2004). Akinetes accumulate glycogen, a storage polysaccharide, and cyanophycin, a nitrogen (N) storage polymer composed of aspartic acid residues with arginine side groups. The DNA content of akinetes can be several times higher than that of vegetative cells. For example, vegetative cells of the filamentous cyanobacterium *Aphanizomenon ovalisporum* were shown to be polyploid with an average of eight copies of the genome per cell while the average number of genome copies in the akinetes of this organism was 119; some of the akinetes had in excess of 400 copies of the genome. Ribosome levels in akinetes were also elevated relative to that of vegetative cells (Sukenik et al., 2012). Akinetes of *Anabaena cylindrica* were also enriched for DNA relative to vegetative cells (Simon, 1977). Interestingly, PPBs were not observed in akinetes but were abundant in vegetative cells, suggesting that polyP in akinetes is used for generating the multiple copies of genomic DNA associated with development, maturation and fruiting of akinetes, and ultimately, the formation of numerous vegetative cells (Sukenik et al., 2012).

### PolyP Accumulation During Sulfur and Nitrogen Deprivation

High levels of polyP can build up in cyanobacteria and algae exposed to stress conditions, including nutrient deprivation and metal ion toxicity (Harold, 1966; Lawry and Jensen, 1979; Lawry and Jensen, 1986; Siderius et al., 1996; Aksoy et al., 2014; Hong-Hermesdorf et al., 2014; Goodenough et al., 2019). In *C. reinhardtii*, polyP accumulation occurs in sulfur (S)-deprived cells and is crucial to allow proper acclimation (Aksoy et al., 2014). In this alga, a mutant lacking one of the subunits of the VTC complex (VTC1) exhibited an aberrant S deprivation response (SdR) with reduced expression of some of S-deprivation-responsive genes. Although the role of polyP in this transcriptional regulation is not well understood, it is known that the inability to synthesize polyP impacts the expression of SdR genes to different degrees. Cells experiencing S starvation induce acclimation in a two-tiered response (Aksoy et al., 2013). Mutants in VTC1 that are unable to accumulate polyP are altered in their abilities to induce genes from both tiers of regulation, with a more significant impact on genes induced during the second tier (*ARSs*, *ECPs*, and *HAPs*) (Aksoy et al., 2014). PolyP accumulation as a consequence of S deprivation has also been described in the green alga *Parachlorella kessleri*, which accumulates polyP during the early stages of starvation, before starch and lipids accumulate (Ota et al., 2016).

PolyP also accumulates in N-deprived algae (Goodenough et al., 2019). An inability to synthesize polyP in the *C. reinhardtii vtc1* mutant impairs accumulation of the L-amino oxidase (LAO1) (Aksoy et al., 2014), a protein that becomes prominent during N deprivation which functions in scavenging ammonium from amino acids. This again highlights a role of the synthesis/accumulation of polyP for normal acclimation to nutrient limitation conditions. The polyP that accumulates during N starvation is degraded and acidocalcisomes resorbed upon exposure of the cells to N replete conditions (Goodenough et al., 2019). PolyP also accumulates in various *Chlorella* strains when they are deprived of N, and becomes the major PO_4_^3-^ source following the addition of N to the cultures (Kuesel et al., 1989; Chu et al., 2013; Chu et al., 2015). When N deprived cells are transferred to N replete medium, polyP is depleted over the first 3 days, even prior to using the PO_4_^3-^ present in the medium. However, when polyP-accumulating cells are exposed to both N and P starvation, they do not use the polyP as a source of PO_4_^3-^. The reason why N is required to use polyP is not clear, but indicates that the absence of extracellular P is not enough to trigger the degradation of polyP and that there is a regulatory hierarchy controlling the induction of nutrient acclimation responses. When cells are deprived only for one nutrient, they induce energy-consuming acclimation responses to optimize the acquisition of that nutrient, facilitate intracellular mobilization, and metabolic adjustment to promote survival. However, if cells experience deprivation for two nutrients, the heirarchy of the evolved cellular responses could favor initiation of only one of the two “programs.” Examining the impact of imposing a limitation for N either prior to or as the cells are exposed to P deprivation (and other multideprivation combinations) may reveal heirarchical responses that reflect the environments in which the organisms evolved.

### PolyP During Stationary Phase and Other Abiotic Stresses

PolyP accumulation has also been linked to osmotic stress and the entrance of specific microbes and phytoplankton into stationary phase (Feuillade et al., 1995; Kornberg et al., 1999; Diaz et al., 2008; Cade-Menun and Paytan, 2010). In *C. reinhardtii*, polyP-filled acidocalcisomes are also generated during stationary phase (Goodenough et al., 2019). Early work with *C. reinhardtii* on polyP showed that one-week-old cultures (stationary phase) grown under mixotrophic conditions generated PPBs that can be released from the cells by exocytosis (Komine et al., 2000). It is not clear whether the accumulation of polyP in stationary phase cells is a consequence of a deficiency for a specific nutrient or to other factors related to the status of the cultures. Similarly, *Chlorella vulgaris* cultures contained little polyP during exponential growth, accumulating it as cells approached stationary phase (Aitchison and Butt, 1973).

PolyP has also been suggested to function in the neutralization of microalgal cells that become alkalinized when incubated in high concentrations of ammonium (Pick et al., 1990). In *Dunaliella salina,* the addition of 20 mM ammonium increased the cytosolic pH and reduced cellular ATP levels. High intracellular pH can dramatically affect photosynthetic and respiratory activities, inhibiting ATP production. When *Dunaliella* cultures experienced high ammonium levels, polyP was degraded and the cells were able to maintain ATP levels and neutralize their cytoplasm; polyP hydrolysis results in H^+^ release, which could compensate for cytsolic alkalinization. In support of these ideas, when algal cells are cultured under conditions of P deprivation and accumulate less polyP, it takes a longer time for them to recover from ammonium “shock.”

Osmotic shock impacts the length of polyP polymers in *Dunaliella salina* and *Phaeodactylum tricornotum* (Bental et al., 1990; Leitao et al., 1995). Hyperosmotic shock promoted the synthesis of longer polymers with concomitant ATP hydrolysis, while the opposite effect, smaller polymer synthesis with an increase in ATP levels, was elicited in cells exposed to hypoosmotic stress. Whether polyP solely functions to maintain the chemical balance in the cell or could have an additional role in the acclimation to omotic stress is unclear.

Some algae accumulate polyP upon exposure to elevated temperatures, but very little is known about this phenomenon in photosynthetic organisms. A *Cylindrocystis*-like alga isolated from the High Arctic, where temperatures oscillate from −12 to +5°C, showed elevated polyP accumulation when cultivated at 20°C (Barcyte et al., 2020). However, another *Cylindrocystis*-like strain that inhabits the desert and grows at elevated temperatures did not accumulate polyP under the same conditions. The different responses of these strains suggest that, as in bacteria, polyP is being synthesized in response to heat stress, and that higher temperatures may be required to elicit its accumulation in the desert alga.

### PolyP as a Chelator of Cations

Several metal ions are critical for growth but become toxic when in excess (*e.g.* Fe, Cu, Zn, Mn, etc.), while other toxic metals ions are nonessential (*e.g.* Cd, Pb, or Hg). Both prokaryotic and eukaryotic photosynthetic organisms have evolved different and sometimes synergistic mechanisms for dealing with toxic levels of metal ions (Twiss and Nalewajko, 1992; Baptista and Vasconcelos, 2006). They can remove the ions from solution by adsorbing them onto their surfaces, import them and sequester them on molecular surfaces within the cell, make functional use of them through biotransformations (*e.g*. incorporation into proteins), increase the activities of efflux pumps, and store them in subcellular compartments (Darnall et al., 1986; Swift and Forciniti, 1997; Baptista and Vasconcelos, 2006).

Several studies have shown that metal ions are bound to the PPBs and sequestered in acidocalcisomes (Baxter and Jensen, 1980; Pettersson et al., 1988; Fiore and Trevors, 1994; Swift and Forciniti, 1997; Docampo et al., 2005; Docampo and Moreno, 2011; Goodenough et al., 2019; Tsednee et al., 2019), with Mg^2+^ and Ca^2+^ being abundant polyP-chelated cations (Siderius et al., 1996; Komine et al., 2000). The presence of Ca^2+^ channels in trypanosomatid acidocalcisome membranes also suggests a possible role of these vacuoles in Ca^2+^ sequestration and exchange, which in turn could impact various signaling pathways (Ramakrishnan and Docampo, 2018). In addition to Ca^2+^ and Mg^2+^, polyP can bind Zn^2+^ Mn^2+^, Al^3+^, and K^+^ and play a role in detoxifying heavy metals such as Cd^2+^ and Pb^2+^. Positive correlations between high levels of polyP and tolerance to cation toxicity have been observed (Sicko-Goad et al., 1975; Baxter and Jensen, 1980; Jensen et al., 1982; Sicko-Goad and Lazinsky, 1986; Torres et al., 1998; Rangsayator et al., 2002; Andrade et al., 2004; Moura et al., 2019), with some cyanobacteria and microalgae also showing increased polyP accumulation during the PO_4_^3-^ overplus reaction when high external metal ion concentrations are included in the medium (Sicko-Goad et al., 1978; Siderius et al., 1996). PolyP levels in the cyanobacteria *Anabaena flos-aquae* and *A*. *variabilis* increased when the cells experienced elevated Zn^2+^ concentrations (Rachlin et al., 1985); a similar increase in polyP was observed when *Spirulina* (*Arthrospira*) *platensis* was exposed to high levels of Cd^2+^ (Rangsayator et al., 2002), when *Plectonema boryanum* was exposed to high levels of Mg^2+^, Sr^2+^, Ba^2+^, and Mn^2+^ redundant (Baxter and Jensen, 1980) and when *Microcystis novacekii* BA005 or *Nostoc paludosum* BA033 were exposed to high Mn^2+^ concentrations (Moura et al., 2019). However, it was also observed that exposure of *C. reinhardtii* to Cd^2+^ and Hg^2+^ elicit polyP degradation and an increase in the amount of short chain polyP and orthophosphate in the vacuoles as the metal ion is being sequestered (Nishikawa et al., 2003; Samadani and Dewez, 2018a; Samadani and Dewez, 2018b), suggesting that the energy in the phosphoanhydride bond and/or the polymer length might contribute to more effective sequestration.

PO_4_³^-^ levels can not only impact cation accumulation, but also the tolerance of photosynthetic microbes to toxic metals. When *C. reinhartii* and *Scenedesmus obliquus* were administered elevated levels of PO_4_³^-^ they accumulated more Cd^2+^ and Zn^2+^ (Yu and Wang, 2004a; Yu and Wang, 2004b) and increases in accumulation of Cu^2+^ and Cd^2+^ in *C. reinhardtii* cells exposed to elevated PO_4_³^-^ concentrations have been correlated with an increase in the tolerance of the cells to the potentially toxic effects of these metal ions (the intracellular stoichiometry of metal ion to PO_4_³^-^ was most important in assessing toxicity) (Wang and Dei, 2006). In the cyanobacterium *M. aeruginosa*, Cd^2+^ and Zn^2+^ uptake was promoted by elevated cellular PO_4_^3-^ concentrations, with short term Cd^2+^ and Zn^2+^ uptake rates increased by 40- and 16-fold, respectively, when the cellular PO_4_^3-^ concentration was increased from 66 to 118 μmol/g dry weight. P-enriched cells also exhibited greater tolerance to Cd^2+^ and Zn^2+^ than cells deprived of P (Zeng and Wang, 2009; Zeng et al., 2009). A mutant of *Nostoc muscorum* that was resistant to Ni^2+^ exhibited a two fold increase in Ni^2+^ uptake relative to the wild type strain, with a concomitant increase in the level of cellular polyP (Singh, 2012). These results all strongly support the idea that many photosynthetic microbes, both prokaryotic and eukaryotic, in an environment with sufficient P can synthesize polyP, mostly as PPBs, that serves as scaffolds for metal binding and detoxification and potentially also as a source of energy for maintaining metal homeostasis.

A similar situation may also occur in some macroalgae. For example, the macroalga *Macrocystis pyrifera* accumulated more Cd^2+^ when it was induced to synthesize high levels of PPBs (Walsh and Hunter, 1992). Elevated capacities to tolerate and sequester metal ions that are potentially toxic, including Pb^2+^ and Cu^2+^, have also been described for organisms of the photosynthetic microbial mat communities of the Ebro Delta in Catalonia (Esteve et al., 1992; Guerrero et al., 1999; Manosa et al., 2001; Burgos et al., 2013). Specific cyanobacteria that live in these microbial communities, including *Oscillatoria* sp. PCC 7515, *Chroococcus* sp. PCC 9106 and *Spirulina* sp. PCC 6313, have high tolerances for Pb^2+^ (Mateo et al., 1997; Maldonado et al., 2010; Maldonado et al., 2011) and have been shown to take up this metal and accumulate it in association with extracellular polymers and intracellular PPBs (Maldonado et al., 2011). A marine *Synechococcus* strain has been shown to sequester radionuclides, including uranium, with much of it bound to polysaccharides on the surface of the cells (Sakaguchi et al., 1978; Acharya et al., 2009). However, an acid soluble polyP may also function in uranium sequestration. Based on energy dispersive X-ray (EDX) spectroscopy and spectrophotometric analyses it was determined that PPBs can form on the surface of the cells of the nitrogen-fixing filamentous cyanobacterium *Anabaena torulosa* and bind uranium (Acharya et al., 2012; Acharya and Apte, 2013).

*C. reinhardtii* represents a robust model microalgal system that is relatively easy to maniputlate at the genetic and molecular levels and has been used to dissect some molecular aspects of metal chelation and toxicity (Hanikenne, 2003). Recent studies have shown that this alga can accumulate Mn^2+^, which colocalizes with polyP and Ca^2+^ in acidocalcisomes (Tsednee et al., 2019). The capacity of *C. reinhardtii* to store Mn^2+^ was also shown to depend on its ability to accumulate polyP. The *vtc1* mutant, which is unable to synthesize polyP, does not accumulate Mn^2+^; the same inability to accumulate Mn^2+^ occurs in wild type cells when they are deprived of P (and cannot make polyP). Interestingly, little of the sequestered Mn^2+^ was chelated directly to polyP, which has led to the suggestion that polyP serves as an intermediate in the binding of Mn^2+^ to a ligand that has not yet been defined. Algal cells, like cyanobacteria (mentioned above), may also exploit extracellular polymers, including polyP (located in the cell wall), as a first line of defense against metal toxicity (Penen et al., 2017). *C. reinhardtii* cell wall-less mutants exhibited reduced growth in the presence of various metals, including Cd^2+^, Co^2+^, Ni^2+^, and Cu^2+^ (Macfie and Welbourne, 2014). This sensitivity could reflect reduced chelation of these metal ions in the absence of extracellular polyP (Werner et al., 2007). Interestingly, both *C. reinhardtii* and *C. acidophila* growing in the presence of heavy metals (*e.g.* Cd^2+^, Hg^2+^) degrades polyP (Nishikawa et al., 2003; Nishikawa et al., 2009; Samadani and Dewez, 2018a; Samadani and Dewez, 2018b), which promotes an increase in the concentration of shorter chains of polyP, free PO_4_^3-^ and ultimately a decrease in intracellular PO_4_^3-^. The mechanism for metal tolerance associated with these changes in polyP features/levels may involve the initial chelation of the toxic metals to polyP which is required for some other form of sequestration that ameliorates metal toxicity; this may relate to the ultimate use of another cation chelator as well as excretion of the ion from the cells. The formation of palmelloid cell aggregates of *C. reinhardtii* cells may also contribute to the cell's ability to withstand metal toxicity (Samadani and Dewez, 2018a; Samadani et al., 2020). In summary, it seems like there are multiple methods for algal and cyanobacterial cells to cope with metal cation toxicity, with some that require chelation by polyP and others for which the mechanism(s) appear to be more complex.

### PolyP as Substrate for Kinases

Specific enzymes that use polyP to phosphorylate sugars have been identified in bacteria, including cyanobacteria, and belong to the family of proteins designated polyP glucokinases (PPGK, polyphosphate–glucose phosphotransferase, EC 2.7.1.63). These proteins use glucose and polyP (and often nucleotides such as ATP) as substrates to catalyze glucose phosphorylation, generating glucose 6-phosphate. PPGKs were first observed in *Mycobacterium phlei* (Szymona, 1957) and in Gram-positive bacteria in the order *Actinomycetales* (Szymona and Ostrowski, 1964; Szymona and Widomski, 1974; Szymona and Szymona, 1978; Szymona and Szymona, 1979; Pepin and Wood, 1986; Mukai et al., 2003; Tanaka et al., 2003; Lindner et al., 2010; Liao et al., 2012; Koide et al., 2013). Although many PPGK enzymes can use a range of nucleotide triphosphates as substrates, the enzymes of the most ancient *Actinomycetales* species have a preference for polyP. In contrast, the more recently evolved bacterial species use ATP and are unable to use polyP, similar to strictly ATP dependent fungal and mammalian hexokinases (Rao et al., 2009). Currently it is thought that the early phosphoryl donor for glucose phosphorylation was polyP and as ATP became the dominant energy donor of the cells, glucokinases evolved into isoforms more specific for ATP, although many of the PPGKs that have been characterized can use both polyP and nucleotide triphosphates as substrates.

A novel subfamily of PPGK proteins have so far, been identified only in N_2_-fixing, filamentous cyanobacteria. Glucokinases from the filamentous nitrogen fixers *Nostoc* sp. PCC7120 (formerly *Anabaena* sp. PCC 7120) and *Nostoc punctiforme* PCC73102 (Klemke et al., 2014; Albi and Serrano, 2015) have been shown to catalyze *in vitro* phosphorylation of glucose using polyP as the phosphoryl donor. These PPGKs have a higher affinity for polyP with more than 10 phosphoryl residues, exhibit moderate activity using mannose as a substrate and, unlike most other glucokinases, appear to be specific for polyP and unable to use nucleoside triphosphates (*e.g.* ATP or GTP) as phosphoryl donors. The gene encoding this enzyme in *Anabaena* sp. PCC 7120 is expressed under conditions of N deprivation and a strain with a null mutation in the gene is compromised under N_2_-fixing conditions (Klemke et al., 2014). It was hypothesized that this glucokinase is part of a response to N deprivation in which “N sparing” reactions (*e.g*. limiting the use of N-containing nucleotides) are elicited.

### Integration of PolyP and Inositol Phosphate Metabolism in Microalgae

PolyP has been associated with active metabolism in various microbes, including algae (Aitchison and Butt, 1973), and can be used as a substrate for the generation of ATP and, in some cases, can phosphorylate other cellular metabolites. As discussed above, its synthesis and degradation are sensitive to environmental conditions and critical for microbes to acclimation to various stress conditions. Therefore, polyP metabolism is linked to the energetics of cells, integral to various cellular processes and likely highly controlled. A link between inositol pyrophosphate metabolism and polyP synthesis has been reported for *Trypanosoma brucei* (Cordeiro et al., 2017) and budding yeast (Wild et al., 2016; Gerasimaite et al., 2017). Mutants in these organisms that are unable to synthesize inositol pyrophosphates (InsP7 and InsP8) exhibited impaired polyP accumulation. In yeast, polyP synthesis has been shown to be activated by the binding of inositol pyrophosphates to the SPX domain of the VTC4 subunit of the VTC complex (Wild et al., 2016). Although the VTC4 protein is currently uncharacterized in algae, based on the genome sequences of various algae, it appears to be conserved in many Chlorophyceae species (e.g., *C. reinhardtii, Chlorella variabilis, Micromonas pusilla, Dunaliella salina,* and *Coccomyxa subellipsoidea,* among others). The VTC4 proteins of the algae contain SPX and polyP polymerase domains, with conservation of specific amino acid residues required for activity (**Figure 1**). These findings suggest that activation of polyP synthesis in microalgae will also respond to inositol pyrophosphates.

Interestingly, a connection between the accumulation of inositol pyrophosphates and TOR signaling has been established in *C. reinhardtii* (Couso et al., 2016). The *vip1* mutant, which is unable to synthesize the highly phosphorylated forms of inositol, exhibited a hypersensitive response to rapamycin (a TOR inhibitor) and more pronounced lipid (triacylglycerol, TAG) synthesis than the wild type strain. Although the precise interactions that link these two pathways and how that link might integrate with polyP metabolism are not understood, the results suggest a connection between polyP and control of cellular energetics, growth, and acclimation through the TOR pathway. Furthermore, the increase in TAG synthesis in inositol pyrophosphate deficient strains indicates that inositol pyrophosphate synthesis and possibly polyP accumulation can impact lipid synthesis and storage. Close interactions between acidocalcisomes and mitochondria observed in *Trypanosoma* and *C. reinhardtii* cells (Docampo and Huang, 2016; Goodenough et al., 2019) also suggests a role (although speculative) for acidocalcisomes and polyP in regulating cellular energetics. The potential integration of polyP metabolism into the central metabolic networks of the cell is an exciting field and may provide new insights into the diversity of phenotypes observed in cells impaired for the synthesis of this polymer.

## PolyP and Biotechnological Applications

Various uses have been proposed for polyP with respect to human health, agricultural practices, and the remediation of ecosystems. PPBs, also recently called “biogenic phosphate nanoparticles” (BPNP), in addition to having roles in PO_4_^3-^ storage, cellular energetics, and metal sequestration, may function in reducing inflammation and protecting mammalian cells from various forms of damage, including oxidative damage (Segawa et al., 2011; Kashima et al., 2015). High levels of BPNPs were synthesized in *Synechococcus* sp PCC7002 overexpressing the the *ppk* gene. The isolated BPNPs from this cyanobacterium were shown to be taken up by polarized human intestinal epithelial (Caco-2) cells (Gao et al., 2018) where they inhibited the induction of nitric oxide synthase and the production of proinflammatory mediators in mouse cell cultures (Feng et al., 2018). This work on potential “health impacts” and uses of polyP for medical applications is intriguing, although it would benefit from further studies of the efficacy and scope of polyP in these processes.

The PPK enzyme can also be used to catalyze ATP production for the synthesis of various compounds. For example, the high temperature resistant PPK from *Thermosynechococcus elongatus* BP-1 was used to generate ATP from ADP and polyP for the production of D-amino acid dipeptide (Sato et al., 2007).

In agriculture, PO_4_^3-^ is an essential nutrient, but its bioavailability in the soil often limits crop productivity. The requirement of PO_4_^3-^ for achieving optimal agricultural yields makes it a critical resource for maintaining food security for future generations. To increase crop yields, high levels of PO_4_^3-^ are often included in fertilizers, although only a small proportion of the applied PO_4_^3-^ may be recovered in the biomass (Pathak et al., 2010); more than 80% of the applied PO_4_^3-^ can be lost in wastewater (*e.g*. parboiled rice mill effluents) or to surface waters as runoff. Hence, in addition to the inefficient economic aspects of applying large amounts of PO_4_^3-^ fertilizer to boost crop productivity, high levels of this nutrient can contaminate agricultural soils, while its runoff into nearby water bodies can cause eutrophication and create toxic habitats (Hupfer et al., 2004; McMahon and Read, 2013). According to several predictions, continued production of high PO_4_^3-^ fertilizers may also become challenging for future generations as the Earth's PO_4_^3-^ reserves might be exhausted in less than 100 years (Cordell and White, 2014; Mukherjee et al., 2015). Furthermore, PO_4_^3–^containing rocks are not equally distributed over the Earth, creating a need for most countries to import this vital nutrient. Indeed, the negative socio-economic impact of limitations in PO_4_^3-^ availability and how, based on current agricultural practices, it can cause environmental degradation, creates a need to reassess the ways in which PO_4_^3-^ is delivered and recycled for the establishment of sustainable agricultural practices (Solovchenko et al., 2016).

To ameliorate the negative impact of amending soils with excess PO_4_^3-^, several studies using cyanobacteria and algae have explored ways to direct polyP metabolism toward bioremediation/water treatment and the production of biofertilizers (Kulakovskaya et al., 2012; Mukherjee et al., 2015; Solovchenko et al., 2016; Siebers et al., 2019). Various cyanobacteria including *Aphanothece* sp., *Spirulina* sp., *Arthrospira* sp., *Lyngbya* sp., *Anabaena* sp., and *Phormidium* sp., as well as eukaryotic microalgae such as *Chlorella* sp. and *Scenedesmus* sp., have been used for removal of nutrients from wastewater (Ray et al., 2013; Mukherjee et al., 2015); much of the excess PO_4_^3-^ that is assimilated by these organisms is polymerized into polyP. Cyanobacteria and microalgae that accumulate high levels of polyP could potentially be integrated into strategies for economical, commercial-scale removal of PO_4_^3-^ from wastewaters and the generation of PO_4_^3–^rich bacterial/algal biomass that could be incorporated into fertilizers for agricultural applications. The rate at which the PO_4_^3-^ is mobilized from these biofertilizers would depend on its degradation/hydrolysis by enzymes released to the soil by microorganisms, with potential slow/moderate release favoring sustained plant growth with reduced PO_4_^3-^ runoff.

The development of polyP-based biological systems for bioremediation and the generation of biofertilizers would benefit from engineered bacterial/algal strains that accumulate excess polyP. The feasibility of developing such strains has been demonstrated for both heterotrophic and photosynthetic bacteria. A high polyP accumulating bacterium was constructed by introducing the *ppk* gene from the cyanobacterium *Microcystis aeruginosa* NIES-843 into *Pseudomonas putida* that was already efficient in removing PO_4_^3-^ from its environment. This strain, grown in a sequencing batch biofilm reactor, had a much higher capacity to remove PO_4_^3-^ from the medium than the control strain (no introduced *ppk* gene) (Du et al., 2012). An alternative approach exploited the finding that mutants in a gene encoding a negative regulatory element of the Pho regulon, PhoU, also accumulated high levels of polyP. Inactivation of the *phoU* gene in *Synechococcus* sp. PCC6803 resulted in elevated intracellular polyP accumulation and a 4-fold increase in the ability of the cells to remove PO_4_^3-^ from the medium relative to the wild type cells (Morohoshi et al., 2002). Furthermore, such strains may also become integral to biostrategies for removing toxic metals from contaminated waters. A more holistic, integrated understanding of how, when, and where polyP is synthesized, as well as the ways in which it interacts with the metabolic and regulatory circuits in the cells, will facilitate the exploitation of its synthesis and accumulation for biotechnological applications.

## Future Perspective

Since the discovery of polyP more than 100 years ago, various functions have been ascribed to this polymer in organisms from all kingdoms of life. PolyP could modulate functionalities of proteins by sequestering cations and influencing the biosynthesis of metalloenzyme and metal toxicity, serve as a scaffold to facilitate protein folding, promote protein degradation, modify proteins through covalent binding (polyphosphorylation), and potentially provide substrate for nucleic acids synthesis. Additionally, polyP can impact Ca^2+^ signaling, contribute to the energy currency of the cell, and impact adenylate homeostasis; these functions can have potential multifactorial effects on a wide variety of cellular processes. Aspects of these functions may guide experimentation toward elucidation of the ways in which this simple molecule assists organisms in coping with environmental challenges.

While our knowledge has slowly accrued over the past half century concerning the synthesis and functions of polyP, there is still little known about the acidocalcisome, which houses polyP, with many questions to be answered. What are the steps in acidocalcisome biogenesis and what makes it different from other vacuoles in the cell? What are the consequences of acidocalcisome interactions with contractile vacuoles and mitochondria and do they interact with other organelles (*e.g.* chloroplasts)? How do acidocalcisomes release their contents (*e.g.* Ca^2+^, polyP, PO_4_^3-^) into the cytoplasm or extracellular space, what signals trigger the release, and how does the release serve ecosystem functions? What role does polyP play in symbiotic interactions between photosynthetic microbes and sponges of the Caribbean coral reef (Zhang et al., 2015), between plants and arbuscular mycorrhiza (Kuga et al., 2008; Tani et al., 2009; Hijikata et al., 2010; Kikuchi et al., 2014; Ferrol et al., 2019), and between algae and animals (Cobb, 1978)? Unraveling the signaling networks that promote release or retainment of polyP granules in phytoplankton will provide a more complete understanding of P cycling and nutrient balancing in nature.

Molecular connections linking the acclimation of cells to stress conditions with polyP metabolism and its impact on cellular energetics and central metabolism, and both the extracellular factors and intracellular regulatory elements that control polyP synthesis, degradation, distribution, and secretion are crucial for elucidating mechanisms by which this ancient molecule modulates cellular processes. This information will also provide the foundational knowledge to foster biotechnological applications that rely on polyP metabolism and to potentially engineer organisms to better cope with adverse environmental conditions, serve in remediation of contaminated ecosystems, and help ameliorate the often detrimental consequences of human activities on our planet.

## Author Contributions

ES-L, DB, and AG discussed and wrote the manuscript.

## Funding

This work was funded by the European Union's Horizon 2020 research and innovation programme under the Marie Sklodowska-Curie grant agreement No. 751039 (to ES-L) and in part by DOE grant DE-SC0008806, which was awarded to AG. DB and AG also acknowledge NSF grant # 1921429.

## Conflict of Interest

The authors declare that the research was conducted in the absence of any commercial or financial relationships that could be construed as a potential conflict of interest.

## Abbreviations

polyP, Polyphosphate; TOR, Target of Rapamycin; PPK, Polyphosphate Kinase; VTC, Vacuolar Transporter Chaperone; Pho (regulon), Phosphate (regulon); V-type ATPase, Vacuolar-type ATPase; H+-PPase, H+-Pyrophosphatase; PPB, Polyphosphate bodies; PPX, Exo-polyphosphatase; TEM, Transmission electron microscopy; SPX domain, Domain conserved in SYG1, Pho81, and XPR11; SdR, Sulfur deprivation responses; PPGK, Polyphosphate glucokinase; InsP, Inositol phosphate; BPNP, Biogenic phosphate nanoparticles.

## References

[B1] Acharya C. Apte S. K. (2013). Novel surface associated polyphosphate bodies sequester uranium in the filamentous, marine cyanobacterium, *Anabaena torulosa* . Metallomics 5, 1595–1598. 10.1039/c3mt00139c 23912813

[B2] Acharya C. Joseph D. Apte S. K. (2009). Uranium sequestration by a marine cyanobacterium, *Synechococcus elongatus* strain BDU/75042. Bioresour. Technol. 100, 2176–2181. 10.1016/j.biortech.2008.10.047 19070485

[B3] Acharya C. Chandwadkar P. Apte S. K. (2012). Interaction of uranium with a filamentous, heterocystous, nitrogen-fixing cyanobacterium, *Anabaena torulosa* . Bioresour. Technol. 116, 290–294. 10.1016/j.biortech.2012.03.068 22522016

[B4] Achbergerova L. Nahalka J. (2011). Polyphosphate–an ancient energy source and active metabolic regulator. Microb. Cell Fact 10, 63. 10.1186/1475-2859-10-63 21816086 PMC3163519

[B5] Adams M. M. Gomez-Garcia M. R. Grossman A. R. Bhaya D. (2008). Phosphorus deprivation responses and phosphonate utilization in a thermophilic *Synechococcus* sp. from microbial mats. J. Bacteriol. 190, 8171–8184. 10.1128/JB.01011-08 18931115 PMC2593230

[B6] Aitchison P. A. Butt V. S. (1973). The relation between the synthesis of inorganic polyphosphate and phosphate uptake by *Chlorella vulgaris* . J. Exp. Bot. 24, 497–510. 10.1093/jxb/24.3.497

[B7] Akiyama M. Crooke E. Kornberg A. (1993). An exopolyphosphatase of *Escherichia coli.* The enzyme and its ppx gene in a polyphosphate operon. J. Biol. Chem. 268, 633–639. 8380170

[B8] Aksoy M. Pootakham W. Pollock S. V. Moseley J. L. Gonzalez-Ballester D. Grossman A. R. (2013). Tiered regulation of sulfur deprivation responses in *Chlamydomonas reinhardtii* and identification of an associated regulatory factor. Plant Physiol. 162, 195–211. 10.1104/pp.113.214593 23482872 PMC3641202

[B9] Aksoy M. Pootakham W. Grossman A. R. (2014). Critical function of a *Chlamydomonas reinhardtii* putative polyphosphate polymerase subunit during nutrient deprivation. Plant Cell 26, 4214–4229. 10.1105/tpc.114.129270 25281687 PMC4247568

[B10] Albi T. Serrano A. (2015). Two strictly polyphosphate-dependent gluco(manno)kinases from diazotrophic cyanobacteria with potential to phosphorylate hexoses from polyphosphates. Appl. Microbiol. Biotechnol. 99, 3887–3900. 10.1007/s00253-014-6184-7 25381489

[B11] Anderson D. M. Cembella A. D. Hallegraeff G. M. (2012). Progress in understanding harmful algal blooms: paradigm shifts and new technologies for research, monitoring, and management. Ann. Rev. Mar. Sci. 4, 143–176. 10.1146/annurev-marine-120308-081121 PMC5373096 22457972

[B12] Andrade L. Keim C. N. Farina M. Pfeiffer W. C. (2004). Zinc detoxification by a cyanobacterium from a metal contaminated bay in Brazil. Braz. Arch. Biol. Biotechnol. 47, 147–152. 10.1590/S1516-89132004000100020

[B13] Andreeva N. Ryazanova L. Dmitriev V. Kulakovskaya T. Kulaev I. (2014). Cytoplasmic inorganic polyphosphate participates in the heavy metal tolerance of *Cryptococcus humicola* . Folia Microbiol. (Praha) 59, 381–389. 10.1007/s12223-014-0310-x 24531869

[B14] Asaoka M. Segami S. Maeshima M. (2014). Identification of the critical residues for the function of vacuolar H(+)-pyrophosphatase by mutational analysis based on the 3D structure. J. Biochem. 156, 333–344. 10.1093/jb/mvu046 25070903

[B15] Aschar-Sobbi R. Abramov A. Y. Diao C. Kargacin M. E. Kargacin G. J. French R. J. (2008). High sensitivity, quantitative measurements of polyphosphate using a new DAPI-based approach. J. Fluoresc. 18, 859–866. 10.1007/s10895-008-0315-4 18210191

[B16] Au K. M. Barabote R. D. Hu K. Y. Saier M. H. Jr. (2006). Evolutionary appearance of H+-translocating pyrophosphatases. Microbiology 152, 1243–1247. 10.1099/mic.0.28581-0 16622041

[B17] Auesukaree C. Tochio H. Shirakawa M. Kaneko Y. Harashima S. (2005). Plc1p, Arg82p, and Kcs1p, enzymes involved in inositol pyrophosphate synthesis, are essential for phosphate regulation and polyphosphate accumulation in Saccharomyces cerevisiae. J. Biol. Chem. 280, 25127–25133. 10.1074/jbc.M414579200 15866881

[B18] Azevedo C. Saiardi A. (2017). Eukaryotic Phosphate Homeostasis: The Inositol Pyrophosphate Perspective. Trends Biochem. Sci. 42, 219–231. 10.1016/j.tibs.2016.10.008 27876550

[B19] Azevedo C. Livermore T. Saiardi A. (2015). Protein polyphosphorylation of lysine residues by inorganic polyphosphate. Mol. Cell 58, 71–82. 10.1016/j.molcel.2015.02.010 25773596

[B20] Baptista M. S. Vasconcelos M. T. (2006). Cyanobacteria metal interactions: Requirements, toxicity and ecological implications. Crit. Rev. Microbiol. 32, 127–137. 10.1080/10408410600822934 16893750

[B21] Barcyte D. Pilatova J. Mojzes P. Nedbalova L. (2020). The Arctic *Cylindrocystis* (Zygnematophyceae, Streptophyta) Green Algae are Genetically and Morphologically Diverse and Exhibit Effective Accumulation of Polyphosphate. J. Phycol. 56, 217–232. 10.1111/jpy.12931 31610035

[B22] Bar-Yosef Y. Sukenik A. Hadas O. Viner-Mozzini Y. Kaplan A. (2010). Enslavement in the water body by toxic *Aphanizomenon ovalisporum*, inducing alkaline phosphatase in phytoplanktons. Curr. Biol. 20, 1557–1561. 10.1016/j.cub.2010.07.032 20705465

[B23] Baxter M. Jensen T. (1980). Uptake of magnesium, strontium, barium and manganese by *Plectonema boryanum* with special reference to polyphosphate bodies. Protoplasma 104, 81–89. 10.1007/BF01279371

[B24] Beauvoit B. Rigoulet M. Guerin B. Canioni P. (1989). Polyphosphates as a source of high energy phosphates in yeast mitochondria: A ^31^P NMR study. FEBS Lett. 252, 17–21. 10.1016/0014-5793(89)80882-8

[B25] Bental M. Pick U. Avron M. Degani H. (1990). Metabolic studies with NMR spectroscopy of the alga *Dunaliella salina* trapped within agarose beads. Eur. J. Biochem. 188, 111–116. 10.1111/j.1432-1033.1990.tb15377.x 2318196

[B26] Besteiro S. Tonn D. Tetley L. Coombs G. H. Mottram J. C. (2008). The AP3 adaptor is involved in the transport of membrane proteins to acidocalcisomes of *Leishmania* . J. Cell Sci. 121, 561–570. 10.1242/jcs.022574 18252798

[B27] Binder B. J. Chisholm S. W. (1990). Relationship between DNA cycle and growth rate in *Synechococcus* sp. strain PCC 6301. J. Bacteriol. 172, 2313–2319. 10.1128/jb.172.5.2313-2319.1990 2110139 PMC208864

[B28] Binder B. J. Chisholm S. W. (1995). Cell Cycle Regulation in Marine *Synechococcus* sp. Strains. Appl. Environ. Microbiol. 61, 708–717. 10.1128/AEM.61.2.708-717.1995 16534938 PMC1388356

[B29] Bjorkman K. M. (2014). Polyphosphate goes from pedestrian to prominent in the marine P-cycle. Proc. Natl. Acad. Sci. U. S. A 111, 7890–7891. 10.1073/pnas.1407195111 24911005 PMC4050537

[B30] Blaby-Haas C. E. Merchant S. S. (2017). Regulating cellular trace metal economy in algae. Curr. Opin. Plant Biol. 39, 88–96. 10.1016/j.pbi.2017.06.005 28672168 PMC5595633

[B31] Braun P. D. Schulz-Vogt H. N. Vogts A. Nausch M. (2018). Differences in the accumulation of phosphorus between vegetative cells and heterocysts in the cyanobacterium *Nodularia spumigena* . Sci. Rep. 8, 5651. 10.1038/s41598-018-23992-1 29618756 PMC5884831

[B32] Brown M. R. Kornberg A. (2004). Inorganic polyphosphate in the origin and survival of species. Proc. Natl. Acad. Sci. U. S. A 101, 16085–16087. 10.1073/pnas.0406909101 15520374 PMC528972

[B33] Brown M. R. Kornberg A. (2008). The long and short of it - polyphosphate, PPK and bacterial survival. Trends Biochem. Sci. 33, 284–290. 10.1016/j.tibs.2008.04.005 18487048

[B34] Bru S. Jimenez J. Canadell D. Arino J. Clotet J. (2016). Improvement of biochemical methods of polyP quantification. Microb. Cell 4, 6–15. 10.15698/mic2017.01.551 28357384 PMC5354550

[B35] Burgos A. Maldonado J. De Los Rios A. Sole A. Esteve I. (2013). Effect of copper and lead on two consortia of phototrophic microorganisms and their capacity to sequester metals. Aquat. Toxicol. 140-141, 324–336. 10.1016/j.aquatox.2013.06.022 23891781

[B36] Cade-Menun B. J. Paytan B. (2010). Nutrient temperature and light stress alter phosphorus and carbon forms in culture-grown algae. Marine Chem. 121, 27–36. 10.1016/j.marchem.2010.03.002

[B37] Castro C. D. Meehan A. J. Koretsky A. P. Domach M. M. (1995). In situ 31P nuclear magnetic resonance for observation of polyphosphate and catabolite responses of chemostat-cultivated *Saccharomyces cerevisiae* after alkalinization. Appl. Environ. Microbiol. 61, 4448–4453. 10.1128/AEM.61.12.4448-4453.1995 8534109 PMC167753

[B38] Chen K. Y. (1999). Study of polyphosphate metabolism in intact cells by 31-P nuclear magnetic resonance spectroscopy Vol. 23 Eds. Schroder H. C. Muller W. E. G. (Berlin, Heidelberg, New York: Springer-Verlag), 241–252. 10.1007/978-3-642-58444-2_13 10448681

[B39] Chopin C. Lehmal H. Halcrow K. (1997). Polyphosphates in the red macroalga *Chondrus crispus* (Rhodophyceae). New Phytol. 135, 587–594. 10.1046/j.1469-8137.1997.00690.x

[B40] Chu F. F. Chu P. N. Cai P. J. Li W. W. Lam P. K. Zeng R. J. (2013). Phosphorus plays an important role in enhancing biodiesel productivity of *Chlorella vulgaris* under nitrogen deficiency. Bioresour. Technol. 134, 341–346. 10.1016/j.biortech.2013.01.131 23517904

[B41] Chu F. F. Shen X. F. Lam P. K. Zeng R. J. (2015). Polyphosphate during the Regreening of *Chlorella vulgaris* under Nitrogen Deficiency. Int. J. Mol. Sci. 16, 23355–23368. 10.3390/ijms161023355 26426008 PMC4632702

[B42] Cobb A. H. (1978). Inorganic polyphosphate involved in the symbiosis between chloroplasts of alga *Codium fragile* and mollusc *Elysia viridis* . Nature 55, 554–555. 10.1038/272554a0

[B43] Cohen A. Perzov N. Nelson H. Nelson N. (1999). A novel family of yeast chaperons involved in the distribution of V-ATPase and other membrane proteins. J. Biol. Chem. 274, 26885–26893. 10.1074/jbc.274.38.26885 10480897

[B44] Cordeiro C. D. Saiardi A. Docampo R. (2017). The inositol pyrophosphate synthesis pathway in *Trypanosoma brucei is* linked to polyphosphate synthesis in acidocalcisomes. Mol. Microbiol. 106, 319–333. 10.1111/mmi.13766 28792096 PMC5630508

[B45] Cordell D. White S. (2014). Life's Bottleneck: Sustaining the World's Phosphorus for a Food Secure Future. Annu. Rev. Environ. Resour. 39, 161–188. 10.1146/annurev-environ-010213-113300

[B46] Couso I. Evans B. S. Li J. Liu Y. Ma F. Diamond S. (2016). Synergism between Inositol Polyphosphates and TOR Kinase Signaling in Nutrient Sensing, Growth Control, and Lipid Metabolism in Chlamydomonas. Plant Cell 28, 2026–2042. 10.1105/tpc.16.00351 27600537 PMC5059802

[B47] Darnall D. W. Greene B. Henzl M. T. Hosea J. M. McPherson R. A. Sneddon J. (1986). Selective recovery of gold and other metal ions from an algal biomass. Environ. Sci. Technol. 20, 206–208. 10.1021/es00144a018 22288814

[B48] Desfougeres Y. Gerasimaite R. U. Jessen H. J. Mayer A. (2016). Vtc5, a Novel Subunit of the Vacuolar Transporter Chaperone Complex, Regulates Polyphosphate Synthesis and Phosphate Homeostasis in Yeast. J. Biol. Chem. 291, 22262–22275. 10.1074/jbc.M116.746784 27587415 PMC5064005

[B49] Diaz J. M. Ingall E. D. (2010). Fluorometric quantification of natural inorganic polyphosphate. Environ. Sci. Technol. 44, 4665–4671. 10.1021/es100191h 20507063

[B50] Diaz J. Ingall E. Benitez-Nelson C. Paterson D. de Jonge M. D. McNulty I. (2008). Marine polyphosphate: a key player in geologic phosphorus sequestration. Science 320, 652–655. 10.1126/science.1151751 18451299

[B51] Diaz J. M. Björkman K. M. Haley S. T. Ingall E. D. Karl D. M. Longo A. F. (2016). Polyphosphate dynamics at Station ALOHA, North Pacific subtropical gyre. Limnol. Oceanogr. 61, 227–239. 10.1002/lno.10206

[B52] Diaz-Troya S. Perez-Perez M. E. Florencio F. J. Crespo J. L. (2008). The role of TOR in autophagy regulation from yeast to plants and mammals. Autophagy 4, 851–865. 10.4161/auto.6555 18670193

[B53] Dijkstra N. Kraal P. Séguret M. J. M. Flores M. R. Gonzalez S. Rijkenberg M. J. A. (2018). Phosphorus dynamics in and below the redoxcline in the Black Sea and implications for phosphorus burial. Geochim. Cosmochim. Acta 222, 685–703. 10.1016/j.gca.2017.11.016

[B54] Docampo R. Huang G. (2016). Acidocalcisomes of eukaryotes. Curr. Opin. Cell Biol. 41, 66–72. 10.1016/j.ceb.2016.04.007 27125677 PMC4983533

[B55] Docampo R. Moreno S. N. (2011). Acidocalcisomes. Cell Calcium 50, 113–119. 10.1016/j.ceca.2011.05.012 21752464 PMC3156361

[B56] Docampo R. de Souza W. Miranda K. Rohloff P. Moreno S. N. (2005). Acidocalcisomes - conserved from bacteria to man. Nat. Rev. Microbiol. 3, 251–261. 10.1038/nrmicro1097 15738951

[B57] Docampo R. Ulrich P. Moreno S. N. (2010). Evolution of acidocalcisomes and their role in polyphosphate storage and osmoregulation in eukaryotic microbes. Philos. Trans. R. Soc. Lond. B. Biol. Sci. 365, 775–784. 10.1098/rstb.2009.0179 20124344 PMC2817225

[B58] Docampo R. (2016). The origin and evolution of the acidocalcisome and its interactions with other organelles. Mol. Biochem. Parasitol. 209, 3–9. 10.1016/j.molbiopara.2015.10.003 26523947 PMC4851929

[B59] Droop M. R. (1973). Some thoughts on nutrient limitation in algae. J. Phycol. 9, 264–273. 10.1111/j.1529-8817.1973.tb04092.x

[B60] Drozdowicz Y. M. Shaw M. Nishi M. Striepen B. Liwinski H. A. Roos D. S. (2003). Isolation and characterization of TgVP1, a type I vacuolar H+-translocating pyrophosphatase from *Toxoplasma gondii.* The dynamics of its subcellular localization and the cellular effects of a diphosphonate inhibitor. J. Biol. Chem. 278, 1075–1085. 10.1074/jbc.M209436200 12411435

[B61] Du H. Yang L. Wu J. Xiao L. Wang X. Jiang L. (2012). Simultaneous removal of phosphorus and nitrogen in a sequencing batch biofilm reactor with transgenic bacteria expressing polyphosphate kinase. Appl. Microbiol. Biotechnol. 96, 265–272. 10.1007/s00253-011-3839-5 22218771

[B62] Dyhrman S. T. Palenik B. (1999). Phosphate stress in cultures and field populations of the dinoflagellate *Prorocentrum minimum* detected by a single-cell alkaline phosphatase assay. Appl. Environ. Microbiol. 65, 3205–3212. 10.1128/AEM.65.7.3205-3212.1999 10388722 PMC91475

[B63] Dyhrman S. T. Ruttenberg K. C. (2006). Presence and regulation of alkaline phosphatase activity in eukaryotic phytoplankton from the coastal ocean: Implications for dissolved organic phosphorus remineralization. Limnol. Oceanogr. 51, 1381–1390. 10.4319/lo.2006.51.3.1381

[B64] Dyhrman S. T. Chappell P. D. Haley S. T. Moffett J. W. Orchard E. D. Waterbury J. B. (2006). Phosphonate utilization by the globally important marine diazotroph *Trichodesmium* . Nature 439, 68–71. 10.1038/nature04203 16397497

[B65] Dyhrman S. T. Jenkins B. D. Rynearson T. A. Saito M. A. Mercier M. L. Alexander H. (2012). The transcriptome and proteome of the diatom *Thalassiosira pseudonana* reveal a diverse phosphorus stress response. PloS One 7, e33768. 10.1371/journal.pone.0033768 22479440 PMC3315573

[B66] Eixler S. Selig U. Karsten U. (2005). Extraction and detection methods for polyphosphate storage in autotrophic planktonic organisms. Hydrobiologia 533, 135–143. 10.1007/s10750-004-2406-9

[B67] Esteve I. Martinez-Alonso M. Mir J. (1992). Distribution, topology and structure of microbial mat communties in Spain. Preliminary studies. Limnetica 8, 185–195.

[B68] Falkner R. Falkner G. (2003). Distinct adaptivity during phosphate uptake by the Cyanobacterium *Anabaena variabilis* reflects information processing about preceding phosphate supply. J. Trace Microprobe Techniques 21, 363–375. 10.1081/TMA-120020271

[B69] Falkner G. Falkner G. (2011). “The Complex Regulation of the Phosphate Uptake System of Cyanobacteria,” in Bioenergetic Processes of Cyanobacteria: From Evolutionary Singularity to Ecological Diversity. Eds. P G. A. Obinger C. Renger G. (Berlin/Heidelberg: Springer), 109–130.

[B70] Fang J. Rohloff P. Miranda K. Docampo R. (2007a). Ablation of a small transmembrane protein of *Trypanosoma brucei* (TbVTC1) involved in the synthesis of polyphosphate alters acidocalcisome biogenesis and function, and leads to a cytokinesis defect. Biochem. J. 407, 161–170. 10.1042/BJ20070612 17635107 PMC2049025

[B71] Fang J. Ruiz F. A. Docampo M. Luo S. Rodrigues J. C. Motta L. S. (2007b). Overexpression of a Zn^2+^-sensitive soluble exopolyphosphatase from *Trypanosoma cruzi* depletes polyphosphate and affects osmoregulation. J. Biol. Chem. 282, 32501–32510. 10.1074/jbc.M704841200 17827150

[B72] Feng G. Dong S. Huang M. Zeng M. Liu Z. Zhao Y. (2018). Biogenic Polyphosphate Nanoparticles from a Marine Cyanobacterium *Synechococcus* sp. PCC 7002: Production, Characterization, and Anti-Inflammatory Properties In Vitro. Mar. Drugs 16, 322–337. 10.3390/md16090322 PMC6163655 30201855

[B73] Ferrol N. C A.-A. Pérez-Tienda J. (2019). Review: Arbuscular mycorrhizas as key players in sustainable plant phosphorus acquisition: An overview on the mechanisms involved. Plant Sci. 280, 441–447. 10.1016/j.plantsci.2018.11.011 30824024

[B74] Feuillade J. Bielicki G. J.-P. Renou J.-P. (1995). ^31^P-NMR study of natural phytoplankton samples. Hydrobiologia 300/301, 391–398. 10.1007/BF00024480

[B75] Fiore M. F. Trevors J. T. (1994). Cell composition and metal tolerance in cyanobacteria. Biometals 7, 83–103. 10.1007/BF00140478

[B76] Fraley C. D. Rashid M. H. Lee S. S. Gottschalk R. Harrison J. Wood P. J. (2007). A polyphosphate kinase 1 (ppk1) mutant of *Pseudomonas aeruginosa* exhibits multiple ultrastructural and functional defects. Proc. Natl. Acad. Sci. U. S. A 104, 3526–3531. 10.1073/pnas.0609733104 17360677 PMC1803759

[B77] Gao F. Wu H. Zeng M. Huang M. Feng G. (2018). Overproduction, purification, and characterization of nanosized polyphosphate bodies from *Synechococcus* sp. PCC 7002. Microb. Cell Fact 17, 27. 10.1186/s12934-018-0870-6 29463242 PMC5819187

[B78] Gerasimaite R. Mayer A. (2016). Enzymes of yeast polyphosphate metabolism: structure, enzymology and biological roles. Biochem. Soc. Trans. 44, 234–239. 10.1042/BST20150213 26862210

[B79] Gerasimaite R. Sharma S. Desfougeres Y. Schmidt A. Mayer A. (2014). Coupled synthesis and translocation restrains polyphosphate to acidocalcisome-like vacuoles and prevents its toxicity. J. Cell Sci. 127, 5093–5104. 10.1242/jcs.159772 25315834

[B80] Gerasimaite R. Pavlovic I. Capolicchio S. Hofer A. Schmidt A. Jessen H. J. (2017). Inositol Pyrophosphate Specificity of the SPX-Dependent Polyphosphate Polymerase VTC. ACS Chem. Biol. 12, 648–653. 10.1021/acschembio.7b00026 28186404

[B81] Ghosh S. Shukla D. Suman K. Lakshmi B. J. Manorama R. Kumar S. (2013). Inositol hexakisphosphate kinase 1 maintains hemostasis in mice by regulating platelet polyphosphate levels. Blood 122, 1478–1486. 10.1182/blood-2013-01-481549 23782934

[B82] Gomes F. M. Ramos I. B. Wendt C. Girard-Dias W. De Souza W. Machado E. A. (2013). New insights into the in situ microscopic visualization and quantification of inorganic polyphosphate stores by 4',6-diamidino-2-phenylindole (DAPI)-staining. Eur. J. Histochem. 57, e34. 10.4081/ejh.2013.e34 24441187 PMC3896036

[B83] Gomes-Vieira A. L. Wideman J. G. Paes-Vieira L. Gomes S. L. Richards T. A. Meyer-Fernandes J. R. (2018). Evolutionary conservation of a core fungal phosphate homeostasis pathway coupled to development in *Blastocladiella emersonii* . Fungal Genet. Biol. 115, 20–32. 10.1016/j.fgb.2018.04.004 29627365

[B84] Gomez-Garcia M. R. Losada M. Serrano A. (2003). Concurrent transcriptional activation of *ppa* and *ppx* genes by phosphate deprivation in the cyanobacterium *Synechocystis* sp. strain PCC 6803. Biochem. Biophys. Res. Commun. 302, 601–609. 10.1016/S0006-291X(03)00162-1 12615077

[B85] Gomez-Garcia M. R. Fazeli F. Grote A. Grossman A. R. Bhaya D. (2013). Role of polyphosphate in thermophilic *Synechococcus* sp. from microbial mats. J. Bacteriol. 195, 3309–3319. 10.1128/JB.00207-13 23687278 PMC3719534

[B86] Goodenough U. Heiss A. A. Roth R. Rusch J. Lee J. H. (2019). Acidocalcisomes: Ultrastructure, biogenesis, and distribution in microbial eukaryotes. Protist 170, 287–313. 10.1016/j.protis.2019.05.001 31154072

[B87] Gray M. J. Wholey W. Y. Wagner N. O. Cremers C. M. Mueller-Schickert A. Hock N. T. (2014). Polyphosphate is a primordial chaperone. Mol. Cell 53, 689–699. 10.1016/j.molcel.2014.01.012 24560923 PMC3996911

[B88] Grillo J. F. Gibson J. (1979). Regulation of phosphate accumulation in the unicellular cyanobacterium *Synechococcus* . J. Bacteriol. 140, 508–517. 10.1128/JB.140.2.508-517.1979 227842 PMC216676

[B89] Guerrero R. Haselton A. Sole M. Margulis L. (1999). *Titanospirillum velox*: A huge, speedy sulfur storing spirillum from Ebro Delta microbial mats. Proc. Natl. Acad. Sci. U.S.A. 96, 11584–11588. 10.1073/pnas.96.20.11584 10500220 PMC18077

[B90] Hanikenne M. (2003). *Chlamydomonas reinhardtii* as a eukaryotic photosynthetic model for studies of heavy metal homeostasis and tolerance. New Phytol. 159, 331–340. 10.1046/j.1469-8137.2003.00788.x 33873346

[B91] Harke M. J. Berry D. L. Ammerman J. W. Gobler C. J. (2012). Molecular response of the bloom-forming cyanobacterium, *Microcystis aeruginosa*, to phosphorus limitation. Microb. Ecol. 63, 188–198. 10.1007/s00248-011-9894-8 21720829

[B92] Harold F. M. (1963). Accumulation of inorganic polyphosphate in *Aerobacter aerogenes.* I. Relationship to growth and nucleic acid synthesis. J. Bacteriol. 86, 216–221. 10.1128/JB.86.2.216-221.1963 14058944 PMC278411

[B93] Harold F. M. (1964). Enzymatic and genetic control of polyphosphate accumulation in *Aerobacter aerogenes* . J. Gen. Microbiol. 35, 81–90. 10.1099/00221287-35-1-81 14171262

[B94] Harold F. M. (1966). Inorganic polyphosphates in biology: structure, metabolism, and function. Bacteriol. Rev. 30, 772–794. 10.1128/MMBR.30.4.772-794.1966 5342521 PMC441015

[B95] Heuser J. E. (2011). The origins and evolution of freeze-etch electron microscopy. J. Electron Microsc. 60, S3–S29. 10.1093/jmicro/dfr044 PMC3202940 21844598

[B96] Hijikata N. Murase M. Tani C. Ohtomo R. Osaki M. Ezawa T. (2010). Polyphosphate has a central role in the rapid and massive accumulation of phosphorus in extraradical mycelium of an arbuscular mycorrhizal fungus. New Phytol. 186, 285–289. 10.1111/j.1469-8137.2009.03168.x 20409186

[B97] Hirani T. A. Suzuki I. Murata N. Hayashi H. Eaton-Rye J. J. (2001). Characterization of a two-component signal transduction system involved in the induction of alkaline phosphatase under phosphate-limiting conditions in *Synechocystis* sp. PCC 6803. Plant Mol. Biol. 45, 133–144. 10.1023/a:1006425214168 11289505

[B98] Hong-Hermesdorf A. Miethke M. Gallaher S. D. Kropat J. Dodani S. C. Chan J. (2014). Subcellular metal imaging identifies dynamic sites of Cu accumulation in Chlamydomonas. Nat. Chem. Biol. 10, 1034–1042. 10.1038/nchembio.1662 25344811 PMC4232477

[B99] Hood R. D. Higgins S. A. Flamholz A. Nichols R. J. Savage D. F. (2016). The stringent response regulates adaptation to darkness in the cyanobacterium *Synechococcus elongatus* . Proc. Natl. Acad. Sci. U. S. A 113, E4867–E4876. 10.1073/pnas.1524915113 27486247 PMC4995992

[B100] Hooley P. Whitehead M. P. Brown M. R. (2008). Eukaryote polyphosphate kinases: is the ‘Kornberg' complex ubiquitous? Trends Biochem. Sci. 33, 577–582. 10.1016/j.tibs.2008.09.007 18938082

[B101] Hoppe H.-G. (2003). Phosphatase activity in the sea. Hydrobiologia 493, 187–200. 10.1023/A:1025453918247

[B102] Hori K. Okamoto J. Tanji Y. Unno H. (2003). Formation, sedimentation and germination properties of *Anabaena* akinetes. Biochem. Eng. J. 14, 67–73. 10.1016/S1369-703X(02)00136-5

[B103] Hothorn M. Neumann H. Lenherr E. D. Wehner M. Rybin V. Hassa P. O. (2009). Catalytic core of a membrane-associated eukaryotic polyphosphate polymerase. Science 324, 513–516. 10.1126/science.1168120 19390046

[B104] Hsu S-H. Hsiao Y.-Y. Liu P.-F. Lin S.-M. Luo Y.-Y. Pan R.-L. (2009). Purification, characterization, and spectral analyses of histidine-tagged vacuolar H^+^-pyrophosphatase expressed in yeast. Bot. Stud. 50, 291–301.

[B105] Huang G. Fang J. Sant'Anna C. Li Z. H. Wellems D. L. Rohloff P. (2011). Adaptor protein-3 (AP-3) complex mediates the biogenesis of acidocalcisomes and is essential for growth and virulence of *Trypanosoma brucei* . J. Biol. Chem. 286, 36619–36630. 10.1074/jbc.M111.284661 21880705 PMC3196089

[B106] Huang G. Bartlett P. J. Thomas A. P. Moreno S. N. Docampo R. (2013). Acidocalcisomes of *Trypanosoma brucei* have an inositol 1,4,5-trisphosphate receptor that is required for growth and infectivity. Proc. Natl. Acad. Sci. U. S. A 110, 1887–1892. 10.1073/pnas.1216955110 23319604 PMC3562765

[B107] Huang G. Ulrich P. N. Storey M. Johnson D. Tischer J. Tovar J. A. (2014). Proteomic analysis of the acidocalcisome, an organelle conserved from bacteria to human cells. PloS Pathog. 10, e1004555. 10.1371/journal.ppat.1004555 25503798 PMC4263762

[B108] Huang R. Wan B. Hultz M. Diaz J. M. Tang Y. (2018). Phosphatase-Mediated Hydrolysis of Linear Polyphosphates. Environ. Sci. Technol. 52, 1183–1190. 10.1021/acs.est.7b04553 29359927

[B109] Huizing M. Helip-Wooley A. Westbroek W. Gunay-Aygun M. Gahl W. A. (2008). Disorders of lysosome-related organelle biogenesis: clinical and molecular genetics. Annu. Rev. Genomics Hum. Genet. 9, 359–386. 10.1146/annurev.genom.9.081307.164303 18544035 PMC2755194

[B110] Hupfer M. Rube B. Schmieder P. (2004). Origin and diagenesis of polyphosphate in lake sediments: A ^31^P-NMR study. Limnol. Oceanogr. 49, 1–10. 10.4319/lo.2004.49.1.0001

[B111] Hupfer M. Gloess S. Grossart H.-P. (2007). Polyphosphate-accumulating microorganisms in aquatic sediments. Aquat. Microbial Ecol. AQUAT MICROB. Ecol. 47, 299–311. 10.3354/ame047299

[B112] Hupfer M. Glöss S. Schmieder P. Grossart H.-P. (2008). Methods for detection and quantification of polyphosphate and polyphosphate accumulating microorganisms in aquatic sediments. Int. Rev. Hydrobiol. 93, 1–30. 10.1002/iroh.200610935

[B113] Ilikchyan I. N. McKay R. M. Zehr J. P. Dyhrman S. T. Bullerjahn G. S. (2009). Detection and expression of the phosphonate transporter gene *phnD in* marine and freshwater picocyanobacteria. Environ. Microbiol. 11, 1314–1324. 10.1111/j.1462-2920.2009.01869.x 19220397

[B114] Jacobson L. Halmann M. Yariv J. (1982). The molecular composition of the volutin granule of yeast. Biochem. J. 201, 473–479. 10.1042/bj2010473 7046728 PMC1163671

[B115] Jasser I. Callieri C. (2017). “Picocyanobacteria: The smallest cell size cyanobacteria,” in Handbook on Cyanobacterial Monitoring and Cytotoxin Analysis. Eds. Meriluoto J. Spoof L. Codd G. A. (Chickester, UK: John Wiley & Son), 19–27.

[B116] Jensen T. E. Sicko L. M. (1974). Phosphate metabolism in blue-green algae. I. Fine structure of the “polyphosphate overplus” phenomenon in *Plectonema boryanum* . Can. J. Microbiol. 20, 1235–1239. 10.1139/m74-190 4371465

[B117] Jensen T. E. Sicko-Goad L. (1976). Aspects of Phosphate Utilization by Blue-Green Algae. p.^pp. USA: US Environmental Protection Agency, Office of Research and Development, Corvallis Environmental Research Laboratory, Corvallis, Oregon.

[B118] Jensen T. E. Baxter M. J. Rachlin M. Jani V. (1982). Uptake of heavy metals by Plectonema boryanum (Cyanophyceae) into cellular components, especially polyphosphate bodies; an X-ray energy dispersive study. Envinron. Poll A27, 119–127. 10.1016/0143-1471(82)90104-0

[B119] Jensen T. E. (1968). Electron microscopy of polyphosphate bodies in a blue-green alga. Nostoc. Pruniforme Arch. Mikrobiol. 62, 144–152. 10.1007/BF00410400

[B120] Jensen T. E. (1993). “Cyanobacterial ultrastructure,” in Ultrastructure of microalgae. Ed. Berne T. (Florida: CRC Press), 8–51.

[B121] Jimenez J. Bru S. Ribeiro M. P. C. Clotet J. (2017). Polyphosphate: popping up from oblivion. Curr. Genet. 63, 15–18. 10.1007/s00294-016-0611-5 27221322

[B122] Juntarajumnong W. Hirani T. A. Simpson J. M. Incharoensakdi A. Eaton-Rye J. J. (2007). Phosphate sensing in *Synechocystis* sp. PCC 6803: SphU and the SphS-SphR two-component regulatory system. Arch. Microbiol. 188, 389–402. 10.1007/s00203-007-0259-0 17541776

[B123] Kajander T. Kellosalo J. Goldman A. (2013). Inorganic pyrophosphatases: One substrate, three mechanisms. FEBS Lett. 587, 1863–1869. 10.1016/j.febslet.2013.05.003 23684653

[B124] Karl D. M. Bjorkman K. M. (2002). “Dynamics of DOP,” in Biochemistry of Marine Dissolved Organic Matter. Eds. Hansell D. A. Carlson C. A. (New York: Elsevier Science), 249–266.

[B125] Karl D. M. (2002). Nutrient dynamics in the deep blue sea. Trends Microbiol. 10, 410–418. 10.1016/S0966-842X(02)02430-7 12217506

[B126] Karl D. M. (2014). Microbially mediated transformations of phosphorus in the sea: New views of an old cycle. Ann. Rev. Mar. Sci. 6, 279–337. 10.1146/annurev-marine-010213-135046 24405427

[B127] Karlsson-Elfgren I. Brunberg A.-K. (2004). The importance of shallow sediments in the recruitmentof *Anabaena* and *Aphanizomenon* (Cyanophyceae). J. Phycol. 40, 831–836. 10.1111/j.1529-8817.2004.04070.x

[B128] Karlsson-Elfgren I. Rengefors K. Gustafsson S. (2004). Factors regulating recruitment from the sediment to the water column in the bloom-forming cyanobacterium *Gleotrichia echinulata* . Freshwater Biol. 49, 265–273. 10.1111/j.1365-2427.2004.01182.x

[B129] Kashima S. Fujiya M. Konishi H. Ueno N. Inaba Y. Moriichi K. (2015). Polyphosphate, an active molecule derived from probiotic *Lactobacillus brevis*, improves the fibrosis in murine colitis. Transl. Res. 166, 163–175. 10.1016/j.trsl.2015.02.002 25766132

[B130] Kaska D. D. Piscopo I. C. Gibor A. (1985). Intracellular calcium redistribution during mating in *Chlamydomonas reinhardii* . Exp. Cell Res. 160, 371–379. 10.1016/0014-4827(85)90183-1 4043249

[B131] Khoshmanesh A. Cook P. L. Wood B. R. (2012). Quantitative determination of polyphosphate in sediments using Attenuated Total Reflectance-Fourier Transform Infrared (ATR-FTIR) spectroscopy and partial least squares regression. Analyst 137, 3704–3709. 10.1039/c2an35289c 22801463

[B132] Kikuchi Y. Hijikata N. Yokoyama K. Ohtomo R. Handa Y. Kawaguchi M. (2014). Polyphosphate accumulation is driven by transcriptome alterations that lead to near-synchronous and near-equivalent uptake of inorganic cations in an arbuscular mycorrhizal fungus. New Phytol. 204, 638–649. 10.1111/nph.12937 25039900

[B133] Kim E. J. Zhen R. G. Rea P. A. (1994). Heterologous expression of plant vacuolar pyrophosphatase in yeast demonstrates sufficiency of the substrate-binding subunit for proton transport. Proc. Natl. Acad. Sci. U. S. A 91, 6128–6132. 10.1073/pnas.91.13.6128 8016125 PMC44151

[B134] Kizewski F. Liu Y. T. Morris A. Hesterberg D. (2011). Spectroscopic approaches for phosphorus speciation in soils and other environmental systems. J. Environ. Qual. 40, 751–766. 10.2134/jeq2010.0169 21546661

[B135] Klemke F. Beyer G. Sawade L. Saitov A. Korte T. Maldener I. (2014). All1371 is a polyphosphate-dependent glucokinase in *Anabaena* sp. PCC 7120. Microbiology 160, 2807–2819. 10.1099/mic.0.081836-0 25320362 PMC4252912

[B136] Klompmaker S. H. Kohl K. Fasel N. Mayer A. (2017). Magnesium uptake by connecting fluid-phase endocytosis to an intracellular inorganic cation filter. Nat. Commun. 8, 1879. 10.1038/s41467-017-01930-5 29192218 PMC5709425

[B137] Kohl K. Zangger H. Rossi M. Isorce N. Lye L. F. Owens K. L. (2018). Importance of polyphosphate in the Leishmania life cycle. Microb. Cell 5, 371–384. 10.15698/mic2018.08.642 30175107 PMC6116282

[B138] Koide M. Miyanaga A. Kudo F. Eguchi T. (2013). Characterization of polyphosphate glucokinase SCO5059 from *Streptomyces coelicolor* A3(2). Biosci. Biotechnol. Biochem. 77, 2322–2324. 10.1271/bbb.130498 24200789

[B139] Kolozsvari B. Parisi F. Saiardi A. (2014). Inositol phosphates induce DAPI fluorescence shift. Biochem. J. 460, 377–385. 10.1042/BJ20140237 24670057

[B140] Kolozsvari B. Firth S. Saiardi A. (2015). Raman Spectroscopy Detection of Phytic Acid in Plant Seeds Reveals the Absence of Inorganic Polyphosphate. Mol. Plant 8, 826–828. 10.1016/j.molp.2015.01.015 25620771

[B141] Komine Y. Eggink L. L. Park H. Hoober J. K. (2000). Vacuolar granules in *Chlamydomonas reinhardtii*: polyphosphate and a 70-kDa polypeptide as major components. Planta 210, 897–905. 10.1007/s004250050695 10872220

[B142] Kornberg A. Fraley C. D. (2000). Inorganic polyphosphate. A molecular fossil come to light. ASM News 66, 275–280.

[B143] Kornberg A. Kornberg S. R. Simms E. S. (1956). Metaphosphate synthesis by an enzyme from *Escherichia coli* . Biochim. Biophys. Acta 20, 215–227. 10.1016/0006-3002(56)90280-3 13315368

[B144] Kornberg A. Rao N. N. Ault-Riche D. (1999). Inorganic polyphosphate: a molecule of many functions. Annu. Rev. Biochem. 68, 89–125. 10.1146/annurev.biochem.68.1.89 10872445

[B145] Kornberg A. (1995). Inorganic polyphosphate: toward making a forgotten polymer unforgettable. J. Bacteriol. 177, 491–496. 10.1128/JB.177.3.491-496.1995 7836277 PMC176618

[B146] Kuesel A. C. Sianoudis J. Leibfritz D. Grimme L. H. Mayer A. (1989). P-31 in-vivo NMR investigation on the function of polyphosphates as phosphate-and energysource during the regreening of the green alga *Chlorella fusca* . Arch. Microbiol. 152, 167–171. 10.1007/BF00456096

[B147] Kuga Y. Saito K. Nayuki K. Peterson R. L. Saito M. (2008). Ultrastructure of rapidly frozen and freeze-substituted germ tubes of an arbuscular mycorrhizal fungus and localization of polyphosphate. New Phytol. 178, 189–200. 10.1111/j.1469-8137.2007.02345.x 18194149

[B148] Kulaev I. S. Vagabov V. M. Kulakovskaya T. V. (2004). The biochemistry of inorganic polyphosphates. (Chinchester, West Sussex, England: Jon Wiley & Sons, Ltd).

[B149] Kulakovskaya T. Vagabov V. Kulaev I. (2012). Inorganic Polyphosphate in Industry, Agriculture and Medicine: Modern State and Outlook. Process Biochem. 47, 1–10. 10.1016/j.procbio.2011.10.028

[B150] Kuroda A. Murphy H. Cashel M. Kornberg A. (1997). Guanosine tetra- and pentaphosphate promote accumulation of inorganic polyphosphate in *Escherichia coli* . J. Biol. Chem. 272, 21240–21243. 10.1074/jbc.272.34.21240 9261133

[B151] Lahti R. (1983). Microbial inorganic pyrophosphatases. Microbiol. Rev. 47, 169–178. 10.1128/MMBR.47.2.169-178.1983 6135978 PMC281570

[B152] Lander N. Ulrich P. N. Docampo R. (2013). *Trypanosoma brucei* vacuolar transporter chaperone 4 (TbVtc4) is an acidocalcisome polyphosphate kinase required for in vivo infection. J. Biol. Chem. 288, 34205–34216. 10.1074/jbc.M113.518993 24114837 PMC3837161

[B153] Lawry N. H. Jensen T. E. (1979). Deposition of condensed phosphate as an effect of varying sulfur deficiency in the cyanobacterium *Synechococcus* sp. (*Anacystis nidulans*). Arch. Microbiol. 120, 1–7. 10.1007/BF00413264

[B154] Lawry N. H. Jensen T. E. (1986). Condensed phosphate deposition, sulfur amino acid use, and unidirectional transsulfuration in *Synechococcus leopoliensis* . Arch. Microbiol. 144, 317–323. 10.1007/BF00409879

[B155] Leitao J. M. Lorenz B. Bachinski N. Wilhelm C. Muller W. Schroder H. C. (1995). Osmotic stress-induced synthesis and degradation of inorganic polyphosphates in the alga *Phaeodactylum tricornutum* . Mar. Ecol. Prog. Ser. 121, 279–288. 10.3354/meps121279

[B156] Lemercier G. Espiau B. Ruiz F. A. Vieira M. Luo S. Baltz T. (2004). A pyrophosphatase regulating polyphosphate metabolism in acidocalcisomes is essential for *Trypanosoma brucei* virulence in mice. J. Biol. Chem. 279, 3420–3425. 10.1074/jbc.M309974200 14615483

[B157] Li F. J. He C. Y. (2014). Acidocalcisome is required for autophagy in *Trypanosoma brucei* . Autophagy 10, 1978–1988. 10.4161/auto.36183 25484093 PMC4502762

[B158] Liao H. Myung S. Zhang Y.-H. P. (2012). One-step purification and immobilization of thermophilic polyphosphate glucokinase from *Thermobifida fusca* YX: glucose-6-phosphate generation without ATP. Appl. Microbiol. Biotechnol. 93, 1109–1117. 10.1007/s00253-011-3458-1 21766194

[B159] Liberton M. Austin J. Berg R. H. Pakrasi H. B. (2011). Unique thylakoid membrane architecture of a unicellular N_2_-fixing cyanobacterium revealed by electron tomography. Plant Physiol. 155, 1656–1666. 10.1104/pp.110.165332 21173021 PMC3091100

[B160] Lichko L. P. Kulakovskaya T. V. Kulaev I. S. (2010). Properties of partially purified endopolyphosphatase of the yeast *Saccharomyces cerevisiae* . Biochem. (Mosc) 75, 1404–1407. 10.1134/S0006297910110131 21314609

[B161] Lindner S. N. Niederholtmeyer H. Schmitz K. Schoberth S. M. Wendisch V. F. (2010). Polyphosphate/ATP-dependent NAD kinase of *Corynebacterium glutamicum*: biochemical properties and impact of ppnK overexpression on lysine production. Appl. Microbiol. Biotechnol. 87, 583–593. 10.1007/s00253-010-2481-y 20180116

[B162] Liss E. Langen P. (1962). Experiments on polyphosphate overcompensation in yeast cells after phosphate deficiency. Arch. Mikrobiol. 41, 383–392. 10.1007/BF00422195 14465813

[B163] Livermore T. M. Chubb J. R. Saiardi A. (2016a). Developmental accumulation of inorganic polyphosphate affects germination and energetic metabolism in *Dictyostelium discoideum* . Proc. Natl. Acad. Sci. U. S. A 113, 996–1001. 10.1073/pnas.1519440113 26755590 PMC4743807

[B164] Livermore T. M. Azevedo C. Kolozsvari B. Wilson M. S. C. Saiardi A. (2016). Phosphate, inositol and polyphosphates. Biochem. Soc. Trans. 44, 253–259. 10.1042/BST20150215 26862212

[B165] Lonetti A. Szijgyarto Z. Bosch D. Loss O. Azevedo C. Saiardi A. (2011). Identication of an Evolutionarily Conserved Family of Inorganic Polyphosphate Endophosphatases. J. Biol. Chem. 286, 31966–31974. 10.1074/jbc.M111.266320 21775424 PMC3173201

[B166] Lorenzo-Orts L. Couto D. Hothorn M. (2020). Identity and functions of inorganic and inositol polyphosphates in plants. New Phytol. 225, 637–652. 10.1111/nph.16129 31423587 PMC6973038

[B167] Luo S. Rohloff P. Cox J. Uyemura S. A. Docampo R. (2004). *Trypanosoma brucei* plasma membrane-type Ca^2+^-ATPase 1 (TbPMC1) and 2 (TbPMC2) genes encode functional Ca^2+^-ATPases localized to the acidocalcisomes and plasma membrane, and essential for Ca^2+^ homeostasis and growth. J. Biol. Chem. 279, 14427–14439. 10.1074/jbc.M309978200 14724285

[B168] Macfie S. M. Welbourne P. M. (2014). The cell wall as a barrier to the uptake of metal ions in the unicellular green alga *Chlamydomonas reinhardtii* . Arch. Environ. Contam. Toxicol. 39, 413–419. 10.1007/s002440010122 11031300

[B169] Madeira da Silva L. Beverley S. M. (2010). Expansion of the target of rapamycin (TOR) kinase family and function in Leishmania shows that TOR3 is required for acidocalcisome biogenesis and animal infectivity. Proc. Natl. Acad. Sci. U.S.A. 107, 11965–11970. 10.1073/pnas.1004599107 20551225 PMC2900677

[B170] Maldonado J. de los Rios A. Esteve I. Ascaso C. Puyen Z. M. Brambilla C. (2010). Sequestration and in vivo effect of lead on DE2009 microalga, using high-resolution microscopic techniques. J. Hazard Mater. 183, 44–50. 10.1016/j.jhazmat.2010.06.085 20675042

[B171] Maldonado J. Sole A. Puyen Z. M. Esteve I. (2011). Selection of bioindicators to detect lead pollution in Ebro delta microbial mats, using high-resolution microscopic techniques. Aquat. Toxicol. 104, 135–144. 10.1016/j.aquatox.2011.04.009 21570936

[B172] Manganelli R. (2007). Polyphosphate and stress response in mycobacteria. Mol. Microbiol. 65, 258–260. 10.1111/j.1365-2958.2007.05819.x 17590232

[B173] Manosa S. Mateo R. Guitart R. (2001). A review of the effects of agricultural and industrial contamination in the Ebro delta biota and wildlife. Envinron. Monit. Assess. 71, 187–205. 10.1023/A:1017545932219 11686200

[B174] Martin P. Van Mooy B. A. (2013). Fluorometric quantification of polyphosphate in environmental plankton samples: extraction protocols, matrix effects, and nucleic acid interference. Appl. Environ. Microbiol. 79, 273–281. 10.1128/AEM.02592-12 23104409 PMC3536087

[B175] Martin P. Van Mooy B. A. (2015). Correction for martin and van mooy, fluorometric quantification of polyphosphate in environmental plankton samples: extraction protocols, matrix effects, and nucleic acid interference. Appl. Environ. Microbiol. 81, 461. 10.1128/AEM.03436-14 23104409 PMC3536087

[B176] Martin P. Dyhrman S. T. Lomas M. W. Poulton N. J. Van Mooy B. A. (2014). Accumulation and enhanced cycling of polyphosphate by Sargasso Sea plankton in response to low phosphorus. Proc. Natl. Acad. Sci. U. S. A 111, 8089–8094. 10.1073/pnas.1321719111 24753593 PMC4050623

[B177] Martin P. Lauro F. M. Sarkar A. Goodkin N. Prakash P. N. Vinayachandran P. N. (2018). Particulate polyphosphate and alkaline phosphatase activity across a latitudinal transect in the tropical Indian Ocean. Limnol. Oceanogr. 63, 1395–1406. 10.1002/lno.10780

[B178] Martinez R. J. (1963). On the nature of the granules of the genus *Spirillum* . Archiv. Fur Mikrobiol. 4, 334–343. 10.1007/BF00509002

[B179] Mateo R. Martinez-Vilalta A. Guitart R. (1997). Lead shot pellets in the Ebro delta Spain: densities in sediments and prevalence of exposure in waterflow. Environ. Pollut. 96, 335–341. 10.1016/S0269-7491(97)00046-8 15093399

[B180] McIntosh M. T. Vaidya A. B. (2002). Vacuolar type H+ pumping pyrophosphatases of parasitic protozoa. Int. J. Parasitol. 32, 1–14. 10.1016/S0020-7519(01)00325-3 11796117

[B181] McMahon K. D. Read E. K. (2013). Microbial contributions to phosphorus cycling in eutrophic lakes and wastewater. Annu. Rev. Microbiol. 67, 199–219. 10.1146/annurev-micro-092412-155713 23799816

[B182] Meyer A. (1904). Orientierende untersuchungen ueber verbreitung. morphologie, und chemie des volutins. Bot. Zeit 62, 113–152.

[B183] Miranda K. de Souza W. Plattner H. Hentschel J. Kawazoe U. Fang J. (2008). Acidocalcisomes in Apicomplexan parasites. Exp. Parasitol. 118, 2–9. 10.1016/j.exppara.2007.07.009 17761167

[B184] Montalvetti A. Rohloff P. Docampo R. (2004). A functional aquaporin co-localizes with the vacuolar proton pyrophosphatase to acidocalcisomes and the contractile vacuole complex of *Trypanosoma cruzi* . J. Biol. Chem. 279, 38673–38682. 10.1074/jbc.M406304200 15252016

[B185] Moreno S. N. Docampo R. (2009). The role of acidocalcisomes in parasitic protists. J. Eukaryot. Microbiol. 56, 208–213. 10.1111/j.1550-7408.2009.00404.x 19527347 PMC2802266

[B186] Moreno B. Rodrigues C. O. Bailey B. N. Urbina J. A. Moreno S. N. Docampo R. (2002). Magic-angle spinning (31)P NMR spectroscopy of condensed phosphates in parasitic protozoa: visualizing the invisible. FEBS Lett. 523, 207–212. 10.1016/S0014-5793(02)02977-0 12123833

[B187] Moreno-Sanchez D. Hernandez-Ruiz L. Ruiz F. A. Docampo R. (2012). Polyphosphate is a novel pro-inflammatory regulator of mast cells and is located in acidocalcisomes. J. Biol. Chem. 287, 28435–28444. 10.1074/jbc.M112.385823 22761438 PMC3436523

[B188] Mori T. Binder B. Johnson C. H. (1996). Circadian gating of cell division in cyanobacteria growing with average doubling times of less than 24 hours. Proc. Natl. Acad. Sci. U. S. A 93, 10183–10188. 10.1073/pnas.93.19.10183 8816773 PMC38358

[B189] Morohoshi T. Maruo T. Shirai Y. Kato J. Ikeda T. Takiguchi N. (2002). Accumulation of inorganic polyphosphate in phoU mutants of *Escherichia coli* and *Synechocystis* sp. strain PCC6803. Appl. Environ. Microbiol. 68, 4107–4110. 10.1128/AEM.68.8.4107-4110.2002 12147514 PMC124021

[B190] Morrissey J. H. (2012). Polyphosphate multi-tasks. J. Thromb. Haemostasis 10, 2313–2314. 10.1111/jth.12001 23006797 PMC4862873

[B191] Moseley J. Grossman A. R. (2009). “Phosphorus limitation from the physiological to the genomic,” in The Chlamydomonas Sourcebook, vol. 2 . Eds. Harris E. Stern D. Witman G. B. (Elsevier: Academic Press), 189–216.

[B192] Moudrikova S. Sadowsky A. Metzger S. Nedbal L. Mettler-Altmann T. Mojzes P. (2017). Quantification of Polyphosphate in Microalgae by Raman Microscopy and by a Reference Enzymatic Assay. Anal. Chem. 89, 12006–12013. 10.1021/acs.analchem.7b02393 29099580

[B193] Moura K. Lizieri C. Franco M. Vaz M. Araujo W. Convey P. (2019). Physiological and thylakoid ultrastructural changes in cyanobacteria in response to toxic manganese concentrations. Ecotoxicology 28 (8), 1009–1021. 10.1007/s10646-019-02098-y 31471822

[B194] Mukai T. Kawai S. Matsukawa H. Matuo Y. Murata K. (2003). Characterization and molecular cloning of a novel enzyme, inorganic polyphosphate/ATP-glucomannokinase, of *Arthrobacter* sp. strain KM. Appl. Environ. Microbiol. 69, 3849–3857. 10.1128/AEM.69.7.3849-3857.2003 12839753 PMC165190

[B195] Mukherjee C. Chowdhury R. Ray K. (2015). Phosphorus Recycling from an Unexplored Source by Polyphosphate Accumulating Microalgae and Cyanobacteria-A Step to Phosphorus Security in Agriculture. Front. Microbiol. 6, 1421. 10.3389/fmicb.2015.01421 26733966 PMC4686675

[B196] Muller F. Mutch N. J. Schenk W. A. Smith S. A. Esterl L. Spronk H. M. (2009). Platelet polyphosphates are proinflammatory and procoagulant mediators in vivo. Cell 139, 1143–1156. 10.1016/j.cell.2009.11.001 20005807 PMC2796262

[B197] Munoz-Martin M. A. Martinez-Rosell A. Perona E. Fernandez-Pinas F. Mateo P. (2014). Monitoring bioavailable phosphorus in lotic systems: a polyphasic approach based on cyanobacteria. Sci. Total Environ. 475, 158–168. 10.1016/j.scitotenv.2013.06.076 23870499

[B198] Murata K. Hagiwara S. Kimori Y. Kaneko Y. (2016). Ultrastructure of compacted DNA in cyanobacteria by high-voltage cryo-electron tomography. Sci. Rep. 6, 34934. 10.1038/srep34934 27731339 PMC5059737

[B199] Nierzwicki-Bauer S. A. Balkwill D. L. Stevens S. E. Jr. (1983). Three-dimensional ultrastructure of a unicellular cyanobacterium. J. Cell Biol. 97, 713–722. 10.1083/jcb.97.3.713 6411738 PMC2112578

[B200] Nishikawa K. Yamakoshi Y. Uemura I. Tominaga N. (2003). Ultrastructural changes in *Chlamydomonas acidophila* (Chlorophyta) induced by heavy metals and polyphosphate metabolism. FEMS Microbiol. Ecol. 44, 253–259. 10.1016/S0168-6496(03)00049-7 19719642

[B201] Nishikawa K. Machida H. Yamakoshi Y. Ohtomo R. Saito K. Saito M. (2006). Polyphosphate metabolism in an acidophilic alga *Chlamydomonas acidophilia* KT-1 (Chlorophyte) under phosphate stress. Plant Sci. 170, 307–313. 10.1016/j.plantsci.2005.08.025

[B202] Nishikawa K. Tominaga N. Uchino T. Oikawa A. Tokunaga H. (2009). “Polyphosphate contributes to CD tolerance in *Chlamydomonas Acidophila* KT-1,” in Algae: Nutrition, Pollution Control and Energy Sources. Ed. Hagen K. N. (Hauppauge, NY: Nova Science Publishers), 13–21.

[B203] Nitta K. Nagayama K. Danev R. Kaneko Y. (2009). Visualization of BrdU-labelled DNA in cyanobacterial cells by Hilbert differential contrast transmission electron microscopy. J. Microsc. 234, 118–123. 10.1111/j.1365-2818.2009.03162.x 19397740

[B204] Niyogi S. Jimenez V. Girard-Dias W. de Souza W. Miranda K. Docampo R. (2015). Rab32 is essential for maintaining functional acidocalcisomes, and for growth and infectivity of *Trypanosoma cruzi* . J. Cell Sci. 128, 2363–2373. 10.1242/jcs.169466 25964650 PMC4487017

[B205] Ohbayashi R. Watanabe S. Kanesaki Y. Narikawa R. Chibazakura T. Ikeuchi M. (2013). DNA replication depends on photosynthetic electron transport in cyanobacteria. FEMS Microbiol. Lett. 344, 138–144. 10.1111/1574-6968.12166 23621483

[B206] Orchard E. D. Benitez-Nelson C. R. Pellechia P. J. Lomas M. W. Dyhrman S. T. (2010). Polyphosphate in *Trichodesmium* from the low-phosphorus Sargasso Sea. Limnol. Oceanogr. 55, 2161–2169. 10.4319/lo.2010.55.5.2161

[B207] Oren N. Raanan H. Kedem I. Turjeman A. Bronstein M. Kaplan A. (2019). Desert cyanobacteria prepare in advance for dehydration and rewetting: The role of light and temperature sensing. Mol. Ecol. 28, 2305–2320. 10.1111/mec.15074 31025457

[B208] Ota S. Yoshihara M. Yamazaki T. Takeshita T. Hirata A. Konomi M. (2016). Deciphering the relationship among phosphate dynamics, electron-dense body and lipid accumulation in the green alga *Parachlorella kessleri* . Sci. Rep. 6, 25731. 10.1038/srep25731 27180903 PMC4867602

[B209] Pathak H. Mohanty S. Jain N. Bhatia A. (2010). Nitrogen, phosphorus, and potassium budgets in Indian agriculture. Nutr. Cycling Agroecosyst 86, 287–299. 10.1007/s10705-009-9292-5

[B210] Penen F. Malherbe J. Isaure M. P. Dobritzsch D. Bertalan I. Gontier E. (2016). Chemical bioimaging for the subcellular localization of trace elements by high contrast TEM, TEM/X-EDS, and NanoSIMS. J. Trace Elem. Med. Biol. 37, 62–68. 10.1016/j.jtemb.2016.04.014 27288221

[B211] Penen F. Isaure M. P. Dobritzsch D. Bertalan I. Castillo-Michel H. Proux O. (2017). Pools of cadmium in *Chlamydomonas reinhardtii* revealed by chemical imaging and XAS spectroscopy. Metallomics 9, 910–923. 10.1039/C7MT00029D 28598481

[B212] Pepin C. A. Wood H. G. (1986). Polyphosphate glucokinase from *Propionibacterium shermanii.* Kinetics and demonstration that the mechanism involves both processive and nonprocessive type reactions. J. Biol. Chem. 261, 4476–4480. 3007458

[B213] Perry M. J. (1976). Phosphate utilization by an oceanic diatom in phosphorus limited chemostat culture and in oligotrophic waters of the central North Pacific. Limnol. Oceanogr. 21, 88–107. 10.4319/lo.1976.21.1.0088

[B214] Pettersson A. Hallbom L. Bergman B. (1988). Aluminum Effects on Uptake and Metabolism of Phosphorus by the Cyanobacterium *Anabaena cylindrica* . Plant Physiol. 86, 112–116. 10.1104/pp.86.1.112 16665849 PMC1054438

[B215] Pick U. Bental M. Chitlaru E. Weiss M. (1990). Polyphosphate-hydrolysis - a protective mechanism against alkaline stress. FEBS Lett. 274, 15–18. 10.1016/0014-5793(90)81318-I 2253767

[B216] Raboy V. (2003). myo-Inositol-1,2,3,4,5,6-hexakisphosphate. Phytochemistry 64, 1033–1043. 10.1016/S0031-9422(03)00446-1 14568069

[B217] Rachlin J. W. Jensen T. E. Warkentine B. E. (1985). Morphometric analysis of the response of Anabaena flos-aquae and *Anabaena variabilis* (Cyanophyceae) to selected concentrations of zinc. Arch. Environ. Contam. Toxicol. 14, 395–402. 10.1007/BF01055524

[B218] Ramakrishnan S. Docampo M. (2018). Membrane proteins in trypanosomatids involved in Ca^2+^ homeostasis and signaling. Genes 9, 304. 10.3390/genes9060304 PMC6027440 29921754

[B219] Rangsayator N. Upatham E. S. Kruatrachue M. Pokethitiyook P. Lanza G. R. (2002). Phytoremediation potential of *Spirulina* (Arthrospira) *platensis*: biosorption and toxicity studies of cadmium. Environ. Pollut. 119, 45–53. 10.1016/S0269-7491(01)00324-4 12125728

[B220] Rao N. N. Kornberg A. (1999). Inorganic polyphosphate regulates responses of *Escherichia coli to* nutritional stringencies, environmental stresses and survival in the stationary phase. Prog. Mol. Subcell Biol. 23, 183–195. 10.1007/978-3-642-58444-2_9 10448677

[B221] Rao N. N. Gomez-Garcia M. R. Kornberg A. (2009). Inorganic polyphosphate: essential for growth and survival. Annu. Rev. Biochem. 78, 605–647. 10.1146/annurev.biochem.77.083007.093039 19344251

[B222] Rashid M. H. Rumbaugh K. Passador L. Davies D. G. Hamood A. N. Iglewski B. H. (2000). Polyphosphate kinase is essential for biofilm development, quorum sensing, and virulence of *Pseudomonas aeruginosa* . Proc. Natl. Acad. Sci. U. S. A 97, 9636–9641. 10.1073/pnas.170283397 10931957 PMC16917

[B223] Ray K. Mukherjee C. Ghosh A. N. (2013). A way to curb phosphorus toxicity in the environment: use of polyphosphate reservoir of cyanobacteria and microalga as a safe alternative phosphorus biofertilizer for Indian agriculture. Environ. Sci. Technol. 47, 11378–11379. 10.1021/es403057c 24093750

[B224] Rea P. A. Kim Y. Sarafian V. Poole R. J. Davies J. M. Sanders D. (1992). Vacuolar H(+)-translocating pyrophosphatases: a new category of ion translocase. Trends Biochem. Sci. 17, 348–353. 10.1016/0968-0004(92)90313-X 1329278

[B225] Reistetter E. N. Krumhardt K. Callnan K. Roache-Johnson K. Saunders J. K. Moore L. R. (2013). Effects of phosphorus starvation versus limitation on the marine cyanobacterium *Prochlorococcus* MED4 II: gene expression. Environ. Microbiol. 15, 2129–2143. 10.1111/1462-2920.12129 23647921

[B226] Reusch R. N. (1999). Polyphosphate/poly-(R)-3-hydroxybutyrate) ion channels in cell membranes. Prog. Mol. Subcell Biol. 23, 151–182. 10.1007/978-3-642-58444-2_8 10448676

[B227] Reusch R. N. (2000). Transmembrane ion transport by polyphosphate/poly-(R)-3-hydroxybutyrate complexes. Biochem. (Mosc) 65, 280–295. 10739470

[B228] Rhee G.-Y. (1973). A continuous culture study of phosphate uptake, growth rate and polyphosphate in *Scenedesmus* sp. J. Phycol. 9, 495–506.

[B229] Riegman R. Stolte W. Noordeloos A. A. M. Slezak D. (2000). Nutrient uptake, and alkaline phosphate (EC 3 : 1 : 3 : 1) activity of *Emiliania huxleyi* (Prymnesiophyceae) during growth under N and P limitation in continuous cultures. J. Phycol. 36, 87–96. 10.1046/j.1529-8817.2000.99023.x 29544012

[B230] Rodrigues C. O. Scott D. A. Docampo R. (1999). Characterization of a vacuolar pyrophosphatase in *Trypanosoma brucei* and its localization to acidocalcisomes. Mol. Cell Biol. 19, 7712–7723. 10.1128/MCB.19.11.7712 10523660 PMC84816

[B231] Rodrigues C. O. Ruiz F. A. Vieira M. Hill J. E. Docampo R. (2002). An acidocalcisomal exopolyphosphatase from *Leishmania major* with high affinity for short chain polyphosphate. J. Biol. Chem. 277, 50899–50906. 10.1074/jbc.M208940200 12393865

[B232] Ruiz F. A. Marchesini N. Seufferheld M. Govindjee Docampo R. (2001). The polyphosphate bodies of *Chlamydomonas reinhardtii* possess a proton-pumping pyrophosphatase and are similar to acidocalcisomes. J. Biol. Chem. 276, 46196–46203. 10.1074/jbc.M105268200 11579086

[B233] Ruiz F. A. Lea C. R. Oldfield E. Docampo R. (2004a). Human platelet dense granules contain polyphosphate and are similar to acidocalcisomes of bacteria and unicellular eukaryotes. J. Biol. Chem. 279, 44250–44257. 10.1074/jbc.M406261200 15308650

[B234] Ruiz F. A. Luo S. Moreno S. N. Docampo R. (2004b). Polyphosphate content and fine structure of acidocalcisomes of *Plasmodium falciparum* . Microsc. Microanal. 10, 563–567. 10.1017/S1431927604040875 15525430

[B235] Saiardi A. (2012). How inositol pyrophosphates control cellular phosphate homeostasis? Adv. Biol. Regul. 52, 351–359. 10.1016/j.jbior.2012.03.002 22781748

[B236] Sakaguchi T. Horikoshi T. Nakajima A. (1978). Uptake of uranium from sea water by microalgae. J. Fermentation Technol. 56, 561–565.

[B237] Samadani M. Dewez D. (2018a). Effect of mercury on the polyphosphate level of alga *Chlamydomonas reinhardtii* . Environ. Pollut. 240, 506–513. 10.1016/j.envpol.2018.04.141 29754100

[B238] Samadani M. Dewez D. (2018b). Cadmium accumulation and toxicity affect the extracytoplasmic polyphosphate level in *Chlamydomonas reinhardtii* . Ecotoxicol. Environ. Saf. 166, 200–206. 10.1016/j.ecoenv.2018.09.094 30269015

[B239] Samadani M. El-Khoury J. Dewez D. (2020). Tolerance Capacity of Chlamydomonas VHLR Mutants for the Toxicity of Mercury. Water Air Soil Pollut. 231, 167–178. 10.1007/s11270-020-04543-9

[B240] Santos-Beneit F. (2015). The Pho regulon: a huge regulatory network in bacteria. Front. Microbiol. 6, 402. 10.3389/fmicb.2015.00402 25983732 PMC4415409

[B241] Sato M. Masuda Y. Kirimura K. Kino K. (2007). Thermostable ATP regeneration system using polyphosphate kinase from *Thermosynechococcus elongatus* BP-1 for D-amino acid dipeptide synthesis. J. Biosci. Bioeng. 103, 179–184. 10.1263/jbb.103.179 17368402

[B242] Schilling R. K. Tester M. Marschner P. Plett D. C. Roy S. J. (2017). AVP1: One Protein, Many Roles. Trends Plant Sci. 22, 154–162. 10.1016/j.tplants.2016.11.012 27989652

[B243] Scott D. A. Docampo R. (2000). Characterization of isolated acidocalcisomes of *Trypanosoma cruzi* . J. Biol. Chem. 275, 24215–24221. 10.1074/jbc.M002454200 10816577

[B244] Segami S. Asaoka M. Kinoshita S. Fukuda M. Nakanishi Y. Maeshima M. (2018a). Biochemical, Structural and Physiological Characteristics of Vacuolar H+-Pyrophosphatase. Plant Cell Physiol. 59, 1300–1308. 10.1093/pcp/pcy054 29534212

[B245] Segami S. Tomoyama T. Sakamoto S. Gunji S. Fukuda M. Kinoshita S. (2018b). Vacuolar H(+)-Pyrophosphatase and Cytosolic Soluble Pyrophosphatases Cooperatively Regulate Pyrophosphate Levels in Arabidopsis thaliana. Plant Cell 30, 1040–1061. 10.1105/tpc.17.00911 29691313 PMC6002195

[B246] Segawa S. Fujiya M. Konishi H. Ueno N. Kobayashi N. Shigyo T. (2011). Probiotic-derived polyphosphate enhances the epithelial barrier function and maintains intestinal homeostasis through integrin-p38 MAPK pathway. PloS One 6, e23278. 10.1371/journal.pone.0023278 21858054 PMC3156119

[B247] Seki Y. Nitta K. Kaneko Y. (2014). Observation of polyphosphate bodies and DNA during the cell division cycle of *Synechococcus elongatus* PCC 7942. Plant Biol. (Stuttg) 16, 258–263. 10.1111/plb.12008 23574545

[B248] Serrano A. Perez-Castineira J. R. Baltscheffsky M. Baltscheffsky H. (2007). H+-PPases: yesterday, today and tomorrow. IUBMB Life 59, 76–83. 10.1080/15216540701258132 17454298

[B249] Shah N. R. Wilkinson C. Harborne S. P. Turku A. Li K. M. Sun Y. J. (2017). Insights into the mechanism of membrane pyrophosphatases by combining experiment and computer simulation. Struct. Dyn 4, 032105. 10.1063/1.4978038 28345008 PMC5336470

[B250] Shebanova A. Ismagulova T. Solovchenko A. Baulina O. Lobakova E. Ivanova A. (2017). Versatility of the green microalga cell vacuole function as revealed by analytical transmission electron microscopy. Protoplasma 254, 1323–1340. 10.1007/s00709-016-1024-5 27677801

[B251] Shi X. Rao N. N. Kornberg A. (2004). Inorganic polyphosphate in *Bacillus cereus*: motility, biofilm formation, and sporulation. Proc. Natl. Acad. Sci. U. S. A 101, 17061–17065. 10.1073/pnas.0407787101 15572452 PMC535361

[B252] Sicko-Goad L. Jensen T. E. (1976). Phosphate metabolism in blue-green algae. II. Changes in phosphate distribution during starvation and the “polyphosphate overplus” phenomenon in *Plectonema boryanum* . Am. J. Bot. 63, 183–188. 10.1002/j.1537-2197.1976.tb11800.x

[B253] Sicko-Goad L. Lazinsky D. (1986). Quantitative ultrastructural changes associated with lead-coupled luxury phosphate uptake and polyphosphate utilization. Arch. Environ. Contamination Toxicol. 15, 617–627. 10.1007/BF01054908

[B254] Sicko-Goad L. Crang R. E. Jensen T. E. (1975). Phosphate metabolism in blue–green algae. IV.In situ analysis of polyphosphate bodies by X-ray energy dispersive analysis. Cytobiology 11, 430–437.

[B255] Sicko-Goad L. Jensen T. E. Ayala R. P. (1978). Phosphate metabolism in blue-green bacteria. V. Factors affecting phosphate uptake in *Plectonema boryanum* . Can. J. Microbiol. 24, 105–108. 10.1139/m78-019 25703

[B256] Siderius M. Musgrave A. van den Ende H. Koerten H. Cambier P. van der Meer P. (1996). *Chlamydomonas eugametos* (Chlorophyta) stores phosphate in polyphosphate bodies together with calcium. J. Phycol. 32, 402–409. 10.1111/j.0022-3646.1996.00402.x

[B257] Siebers N. Musgrave A. Van den Ende H. Koerten H. Cambier P. Van der Meer P. (2019). Towards phosphorus recycling for agriculture by algae: soil incubation and rhizotron path toward involving microalgae. Algal Res. 43, 101634–101639. 10.1016/j.algal.2019.101634

[B258] Simon R. D. (1977). Macromolecular composition of spores from the filamentous cyanobacterium *Anabaena cylindrica* . J. Bacteriol. 129, 1154–1155. 10.1128/JB.129.2.1154-1155.1977 402350 PMC235059

[B259] Singh A. L. (2012). Study of Ni uptake and its compartmentalization in Ni(r), Pd(r) and Ni(s)/ Pd(s) strain of *Nostoc muscorum* . J. Environ. Sci. Eng. 54, 104–106. 23741865

[B260] Sliwinska-Wilczewska S. Maculewicz J. Barreiro Felpeto A. Latala A. (2018). Allelopathic and Bloom-Forming Picocyanobacteria in a Changing World. Toxins (Basel) 10, 48–67. 10.3390/toxins10010048 PMC5793135 29361682

[B261] Smith R. M. Williams S. B. (2006). Circadian rhythms in gene transcription imparted by chromosome compaction in the cyanobacterium *Synechococcus elongatus* . Proc. Natl. Acad. Sci. U. S. A 103, 8564–8569. 10.1073/pnas.0508696103 16707582 PMC1482530

[B262] Smith S. A. Mutch N. J. Baskar D. Rohloff P. Docampo R. Morrissey J. H. (2006). Polyphosphate modulates blood coagulation and fibrinolysis. Proc. Natl. Acad. Sci. U. S. A 103, 903–908. 10.1073/pnas.0507195103 16410357 PMC1347979

[B263] Solovchenko A. Verschoor A. M. Jablonowski N. D. Nebdal L. (2016). Phosphorus from wastewater to crops: An alternative path involving microalgae. Biotechnol. Adv. 34, 550–564. 10.1016/j.biotechadv.2016.01.002 26795876

[B264] Steinmann M. E. Schmidt R. S. Butikofer P. Maser P. Sigel E. (2017). TbIRK is a signature sequence free potassium channel from *Trypanosoma brucei* locating to acidocalcisomes. Sci. Rep. 7, 656. 10.1038/s41598-017-00752-1 28386071 PMC5429665

[B265] Steunou A. S. Bhaya D. Bateson M. M. Melendrez M. C. Ward D. M. Brecht E. (2006). In situ analysis of nitrogen fixation and metabolic switching in unicellular thermophilic cyanobacteria inhabiting hot spring microbial mats. Proc. Natl. Acad. Sci. U. S. A 103, 2398–2403. 10.1073/pnas.0507513103 16467157 PMC1413695

[B266] Steunou A. S. Jensen S. I. Brecht E. Becraft E. D. Bateson M. M. Kilian O. (2008). Regulation of *nif* gene expression and the energetics of N_2_ fixation over the diel cycle in a hot spring microbial mat. ISME J. 2, 364–378. 10.1038/ismej.2007.117 18323780

[B267] Stevens S. E. Jr. Nierzwicki-Bauer S. A. Balkwill D. L. (1985). Effect of nitrogen starvation on the morphology and ultrastructure of the cyanobacterium *Mastigocladus laminosus* . J. Bacteriol. 161, 1215–1218. 10.1128/JB.161.3.1215-1218.1985 3918986 PMC215029

[B268] Sukenik A. Kaplan-Levy R. N. Welch J. M. Post A. F. (2012). Massive multiplication of genome and ribosomes in dormant cells (akinetes) of *Aphanizomenon ovalisporum* (Cyanobacteria). ISME J. 6, 670–679. 10.1038/ismej.2011.128 21975597 PMC3280138

[B269] Suzuki S. Ferjani A. Suzuki I. Murata N. (2004). The SphS-SphR two component system is the exclusive sensor for the induction of gene expression in response to phosphate limitation in *Synechocystis* . J. Biol. Chem. 279, 13234–13240. 10.1074/jbc.M313358200 14707128

[B270] Swift D. T. Forciniti D. (1997). Accumulation of lead by *Anabaena cylindrica* : mathematical modeling and an energy dispersive X-ray study. Biotechnol. Bioeng. 55, 408–418. 10.1002/(SICI)1097-0290(19970720)55:2<408::AID-BIT18>3.0.CO;2-C 18636499

[B271] Syed Hasnain S. Tanzeelur R. Ghulam Mujtaba S. Syeda Tayyaba B. Saqib Z. (2019). Toxic Algae and Their Environmental Consequences. Trends Telemed. E-Health 1 (4), 1–3. TTEH.000519.002019.

[B272] Szymona M. Ostrowski W. (1964). Inorganic Polyphosphate Glucokinase of *Mycobacterium Phlei* . Biochim. Biophys. Acta 85, 283–295. 10.1016/0926-6569(64)90249-4 14212975

[B273] Szymona O. Szymona M. (1978). Multiple forms of polyphosphate-glucose phosphotransferase in various *Micobacterium* strains. Acta Microbiol. Pol. 27, 73–78. 76428

[B274] Szymona O. Szymona M. (1979). Polyphosphate and ATP-glucose phosphotransferase activities of *Novocardia minima* . Acta Microbiol. Pol. 28, 153–160. 89794

[B275] Szymona M. Widomski J. (1974). A kinetic study on inorganic polyphosphate glucokinase from *Mycobacterium tuberculosis* H37RA. Physiol. Chem. Phys. 6, 393–404. 4217457

[B276] Szymona M. (1957). Utilization of inorganic polyphosphates for phosphorylation of glucose in *Micobacterium phlei* . Bull. Acad. Pol. Sci. Ser. Sci. Biol. 5, 379–381.

[B277] Tanaka S. Lee S. O. Hamaoka K. Kato J. Takiguchi N. Nakamura K. (2003). Strictly polyphosphate-dependent glucokinase in a polyphosphate-accumulating bacterium, *Microlunatus phosphovorus* . J. Bacteriol. 185, 5654–5656. 10.1128/JB.185.18.5654-5656.2003 12949120 PMC193765

[B278] Tani C. Ohtomo R. Osaki M. Kuga Y. Ezawa T. (2009). ATP-dependent but proton gradient-independent polyphosphate-synthesizing activity in extraradical hyphae of an arbuscular mycorrhizal fungus. Appl. Environ. Microbiol. 75, 7044–7050. 10.1128/AEM.01519-09 19767467 PMC2786526

[B279] Tijssen J. P. Beekes H. W. Van Steveninck J. (1982). Localization of polyphosphates in Saccharomyces fragilis, as revealed by 4',6-diamidino-2-phenylindole fluorescence. Biochim. Biophys. Acta 721, 394–398. 10.1016/0167-4889(82)90094-5 7159600

[B280] Torres M. Goldberg J. Jensen T. E. (1998). Heavy metal uptake by polyphosphate bodies in living and killed cells of *Plectonema boryanum* (cyanophycae). Microbios 96, 141–147. 10399343

[B281] Treves H. Raanan H. Finkel O. M. Berkowicz S. M. Keren N. Shotland Y. (2013). A newly isolated Chlorella sp. from desert sand crusts exhibits a unique resistance to excess light intensity. FEMS Microbiol. Ecol. 86, 373–380. 10.1111/1574-6941.12162 23773145

[B282] Treves H. Raanan H. Kedem I. Murik O. Keren N. Zer H. (2016). The mechanisms whereby the green alga Chlorella ohadii, isolated from desert soil crust, exhibits unparalleled photodamage resistance. New Phytol. 210, 1229–1243. 10.1111/nph.13870 26853530

[B283] Tsai J. Y. Kellosalo J. Sun Y. J. Goldman A. (2014). Proton/sodium pumping pyrophosphatases: the last of the primary ion pumps. Curr. Opin. Struct. Biol. 27, 38–47. 10.1016/j.sbi.2014.03.007 24768824

[B284] Tsednee M. Castruita M. Salome P. A. Sharma A. Lewis B. E. Schmollinger S. R. (2019). Manganese co-localizes with calcium and phosphorus in Chlamydomonas acidocalcisomes and is mobilized in manganese-deficient conditions. J. Biol. Chem. 294, 17626–17641. 10.1074/jbc.RA119.009130 31527081 PMC6873200

[B285] Twiss M. R. Nalewajko C. (1992). Influence of phosphorus nutrition on copper toxicity to three strains of *Scenedesmus acuta* (Chlorophyceae). J. Phycol. 28, 291–298. 10.1111/j.0022-3646.1992.00291.x

[B286] Tyrell T. (1999). The influence of nitrogen and phosphorus on oceanic primary production. Nature 400, 525–531. 10.1038/22941

[B287] Ulrich P. N. Lander N. Kurup S. P. Reiss L. Brewer J. Soares Medeiros L. C. (2014). The acidocalcisome vacuolar transporter chaperone 4 catalyzes the synthesis of polyphosphate in insect-stages of *Trypanosoma brucei* and *T. cruzi* . J. Eukaryot. Microbiol. 61, 155–165. 10.1111/jeu.12093 24386955 PMC3959231

[B288] Vaillancourt S. Beuchemin-Newhouse N. Cedergren R. J. (1978). Polyphosphate-deficient mutants of *Anacystis nidulans* . Can. J. Microbiol. 24, 112–116. 10.1139/m78-021 206328

[B289] van Groenestijn J. W. Vlekke G. J. Anink D. M. Deinema M. H. Zehnder A. J. (1988). Role of Cations in Accumulation and Release of Phosphate by Acinetobacter Strain 210A. Appl. Environ. Microbiol. 54, 2894–2901. 10.1128/AEM.54.12.2894-2901.1988 16347788 PMC204401

[B290] Van Mooy B. A. Fredricks H. F. Pedler B. E. Dyhrman S. T. Karl D. M. Koblízek M. (2009). Phytoplankton in the ocean use non-phosphorus lipids in response to phosphorus scarcity. Nature 458, 69–72. 10.1038/nature07659 19182781

[B291] Venter M. Groenewald J. H. Botha F. C. (2006). Sequence analysis and transcriptional profiling of two vacuolar H+ -pyrophosphatase isoforms in *Vitis vinifera* . J. Plant Res. 119, 469–478. 10.1007/s10265-006-0009-4 16924561

[B292] Vercesi A. E. Moreno S. N. Docampo R. (1994). Ca^2+^/^H+^ exchange in acidic vacuoles of *Trypanosoma brucei* . Biochem. J. 304 ( Pt 1), 227–233. 10.1042/bj3040227 7998937 PMC1137476

[B293] Villarreal-Chiu J. F. Quinn J. P. McGrath J. W. (2012). The genes and enzymes of phosphonate metabolism by bacteria, and their distribution in the marine environment. Front. Microbiol. 3, 19. 10.3389/fmicb.2012.00019 22303297 PMC3266647

[B294] Voelz H. Voelz U. Ortigoza R. O. (1966). The “polyphosphate overplus” phenomenon in *Myxococcus xanthus* and its influence on the architecture of the cell. Arch. Mikrobiol. 53, 371–388. 10.1007/BF00409874 5989427

[B295] Voronkov A. Sinetova M. (2019). Polyphosphate accumulation dynamics in a population of *Synechocystis* sp. PCC 6803 cells under phosphate overplus. Protoplasma 256, 1153–1164. 10.1007/s00709-019-01374-2 30972564

[B296] Walsh R. S. Hunter K. A. (1992). Influence of phosphorus storage on the uptake of cadmium by the marine alga *Macrocystis pyrifera* . Limnol. Oceanogr. 37, 1361–1369. 10.4319/lo.1992.37.7.1361

[B297] Wan L. Chen X. Deng Q. Yang L. Li X. Zhang J. (2019). Phosphorus strategy in bloom-forming cyanobacteria (*Dolichospermum* and *Microcystis*) and its role in their succession. Harmful Algae 84, 46–55. 10.1016/j.hal.2019.02.007 31128812

[B298] Wang W.-X. Dei R. C. H. (2006). Metal stoichiometry in predicting Cd and Cu toxicity to a freshwater green alga *Chlamydomonas reinhardtii* . Envinron. Poll 142, 303–312. 10.1016/j.envpol.2005.10.005 16310914

[B299] Wang D.-Z. (2008). Neurotoxins from Marine Dinoflagellates: A Brief Review. Mar. Drugs 6, 349–371. 10.3390/md6020349 18728731 PMC2525493

[B300] Wanner B. L. (1993). Gene regulation by phosphate in enteric bacteria. J. Cell Biochem. 51, 47–54. 10.1002/jcb.240510110 8432742

[B301] Ward D. M. Ferris M. J. Nold S. C. Bateson M. M. (1998). A natural view of microbial biodiversity within hot spring cyanobacterial mat communities. Microbiol. Mol. Biol. Rev. 62, 1353–1370. 10.1128/MMBR.62.4.1353-1370.1998 9841675 PMC98949

[B302] Watanabe S. Ohbayashi R. Shiwa Y. Noda A. Kanesaki Y. Chibazakura T. (2012). Light-dependent and asynchronous replication of cyanobacterial multi-copy chromosomes. Mol. Microbiol. 83, 856–865. 10.1111/j.1365-2958.2012.07971.x 22403820

[B303] Waterbury J. B. Watson S. W. Guillard R. R. L. Brand L. E. (1979). Widespread occurrence of a unicellular, marine, planktonic, cyanobacterium. Nature, 277, 293–294. 10.1038/277293a0

[B304] Weerasekara A. W. Jenkins S. Abbott L. K. Waite I. McGrath J. W. Larma I. (2016). Microbial phylogenetic and functional responses within acidified wastewater communities exhibiting enhanced phosphate uptake. Bioresour. Technol. 220, 55–61. 10.1016/j.biortech.2016.08.037 27566512

[B305] Weisse T. (1993). Dynamics of autotrophic phytoplankton in marine and freshwater ecosystems. Adv. Microbial Ecol., 13, 327–370. 10.1007/978-1-4615-2858-6_8

[B306] Werner T. P. Amrhein N. Freimoser F. M. (2007). Inorganic polyphosphate occurs in the cell wall of *Chlamydomonas reinhardtii* and accumulates during cytokinesis. BMC Plant Biol. 7, 51. 10.1186/1471-2229-7-51 17892566 PMC2096623

[B307] Wild R. Gerasimaite R. Jung J. Y. Truffault V. Pavlovic I. Schmidt A. (2016). Control of eukaryotic phosphate homeostasis by inositol polyphosphate sensor domains. Science 352, 986–990. 10.1126/science.aad9858 27080106

[B308] Xie L. Jakob U. (2018). Inorganic polyphosphate, a multifunctional polyanionic protein scaffold. J. Biol. Chem. 294, 2180–2190. 10.1074/jbc.REV118.002808 30425096 PMC6369292

[B309] Yagisawa F. Nishida K. Yoshida M. Ohnuma M. Shimada T. Fujiwara T. (2009). Identification of novel proteins in isolated polyphosphate vacuoles in the primitive red alga *Cyanidioschyzon merolae* . Plant J. 60, 882–893. 10.1111/j.1365-313X.2009.04008.x 19709388

[B310] Yu R. Q. Wang X.-W. (2004a). Biological uptake of Cd, Se(IV) and Zn by *Chlamydomonas reinhardtii in* response to different phosphate and nitrate additions. Aquat. Microb. Ecol. 35, 163–173. 10.3354/ame035163

[B311] Yu R. Q. Wang X.-W. (2004b). Biokinetics of cadmium, selenium and zinc in freshwater algae *Scenedesmus obliquus* under different phosphorus and nitrogen conditions and metal transfer to *Dafnia magna* . Environ. Pollut. 129, 443–456. 10.1016/j.envpol.2003.11.013 15016465

[B312] Zeng J. Wang W. X. (2009). The importance of cellular phosphorus in controlling the uptake and toxicity of cadmium and zinc in *Microcystis aeruginosa*, a freshwater cyanobacterium. Environ. Toxicol. Chem. 28, 1618–1626. 10.1897/08-639.1 19290679

[B313] Zeng J. Yang L. Wang W. X. (2009). Acclimation to and recovery from cadmium and zinc exposure by a freshwater cyanobacterium, *Microcystis aeruginosa* . Aquat. Toxicol. 93, 1–10. 10.1016/j.aquatox.2009.02.013 19328562

[B314] Zhang F. Blasiak L. C. Karolin J. O. Powell R. J. Geddes C. D. Hill R. T. (2015). Phosphorus sequestration in the form of polyphosphate by microbial symbionts in marine sponges. Proc. Natl. Acad. Sci. U. S. A 112, 4381–4386. 10.1073/pnas.1423768112 25713351 PMC4394258

[B315] Zhu J. Loubery S. Broger L. Zhang Y. Lorenzo-Orts L. Utz-Pugin A. (2020). A genetically validated approach for detecting inorganic polyphosphates in plants. Plant J. 102, 507–576. 10.1111/tpj.14642 31816134

